# A Multigene Phylogeny of Native American Hawkweeds (*Hieracium* Subgen. *Chionoracium*, Cichorieae, Asteraceae): Origin, Speciation Patterns, and Migration Routes

**DOI:** 10.3390/plants11192584

**Published:** 2022-09-30

**Authors:** Judith Fehrer, Yann J. K. Bertrand, Matthias Hartmann, Petra Caklová, Jiřina Josefiová, Siegfried Bräutigam, Jindřich Chrtek

**Affiliations:** 1 Institute of Botany, Czech Academy of Sciences, 25243 Průhonice, Czech Republic; 2 Department of Geobotany & Botanical Garden, Institute of Biology, Martin Luther University Halle-Wittenberg, 06108 Halle (Saale), Germany; 3 Thünen Institute of Biodiversity, Bundesallee 65, 38116 Braunschweig, Germany; 4 Senckenberg Museum für Naturkunde Görlitz, 02826 Görlitz, Germany; 5 Department of Botany, Faculty of Science, Charles University, 12801 Prague, Czech Republic

**Keywords:** *Chionoracium*, molecular dating, molecular markers, *Hieracium*, phylogenetic analysis, *Stenotheca*

## Abstract

Native American hawkweeds are mainly mountainous species that are distributed all over the New World. They are severely understudied with respect to their origin, colonization of the vast distribution area, and species relationships. Here, we attempt to reconstruct the evolutionary history of the group by applying seven molecular markers (plastid, nuclear ribosomal and low-copy genes). Phylogenetic analyses revealed that *Chionoracium* is a subgenus of the mainly Eurasian genus *Hieracium*, which originated from eastern European hawkweeds about 1.58–2.24 million years ago. Plastid DNA suggested a single origin of all *Chionoracium* species. They colonized the New World via Beringia and formed several distinct lineages in North America. Via one Central American lineage, the group colonized South America and radiated into more than a hundred species within about 0.8 million years, long after the closure of the Isthmus of Panama and the most recent uplift of the Andes. Despite some incongruences shown by different markers, most of them revealed the same crown groups of closely related taxa, which were, however, largely in conflict with traditional sectional classifications. We provide a basic framework for further elucidation of speciation patterns. A thorough taxonomic revision of *Hieracium* subgen. *Chionoracium* is recommended.

## 1. Introduction

The geographic origin of taxa, the acquisition of new habitats and subsequent speciation patterns have long intrigued biologists. With the widespread use of molecular markers, deeper insights into these processes have become available, and our understanding of earth’s biodiversity is continuously increasing. Still, considering the enormous richness of forms, among which plants (and animals) are merely better explored than other phyla because they are easier to access morphologically, we have been only scratching the surface in our attempts to classify and understand this diversity. Even in vascular plants, there are still groups that are little explored even though they occupy vast areas. Native American hawkweeds, consisting of approximately 150 species distributed from Alaska to Patagonia, on which we focus in this study, are an example for such an understudied group.

The genus *Hieracium* (Hieraciinae, Cichoriae, Asteraceae) is a large, taxonomically extremely intricate group of herbaceous perennials with its native distribution in Eurasia, North Africa, and America. The number of species worldwide strongly varies, depending on the taxonomic concept (lumping vs. splitting, i.e., collective species vs. microspecies, [1]). Native American hawkweed species were initially separated into *Hieracium* section *Stenotheca* by Torrey and Grey [2]. Later, Fries [3] recognized *Stenotheca* at the rank of a subgenus, which was followed by Peter [4]. However, Schultz [5] had already published the name *Hieracium* subgen. *Chionoracium* for American hawkweed species, and this name therefore supersedes the name *Stenotheca* at the subgenus rank. Besides subgen. *Chionoracium*, *Hieracium* subgen. *Mandonia* was described by Arvet-Touvet [6] based on South American plants. Zahn [7] accepted the two subgenera *Stenotheca* and *Mandonia* for native American hawkweed species in his world monograph of *Hieracium*. The most important changes since Zahn [7] concern the exclusion of the African and European species of sect. *Tolpidiformia* from *Hieracium* [8,9,10,11] and the incorporation of subgen. *Mandonia* into *Chionoracium* [12]. Sell [13], however, suggested that *Chionoracium* belongs to the genus *Crepis*, although the chromosome number and pappi of *Chionoracium* correspond to subgen. *Hieracium*.

The genus *Hieracium* is traditionally divided into three subgenera: *Hieracium*, *Pilosella* and *Chionoracium*. Their basic chromosome number is 2*n* = 18. Subgenus *Hieracium*, with its native range in Eurasia and North America (NA), comprises hundreds to thousands of predominantly triploid or tetraploid species [14], which are (almost) obligate diplosporous apomicts [15,16]. In North America, most species are polyploid and invasive except diploid *H. umbellatum*, which has also the largest distribution area of any diploid *Hieracium* species in Europe, and *H. canadense*, which occurs in northern NA and is sometimes considered a synonym or a subspecies of *H. umbellatum* [17,18]. Subgenus *Pilosella* has been elevated to genus level by Bräutigam and Greuter [19]; it is mainly distributed in Europe and West Asia with about 150 species and comprises a few native species occurring in northwest Africa, but is also introduced to other areas like New Zealand, Australia and America, where it often became invasive [20,21,22]. *Pilosella* is well-known for its extensive variation in ploidy levels, ranging from diploids to octoploids, and facultative aposporous apomixis [23,24]. Subgenus *Hieracium* as well as genus *Pilosella* contain only relatively few sexual diploids (around 20 each, [25,26,27]). Subgenus *Chinoracium*, on which we focus here, consists, as far as known, only of entirely sexual, diploid species [17,28,29,30]. It is native to North and South America and includes 24 species in North America [18], 19 species in Central America [29], and more than 100 species in South America [7,31]. It has a primarily montane distribution; rather few species occur at low elevations. Morphologically, the subgenus can be distinguished by the arrangement and morphology of involucral bracts (a graduated series in *Hieracium*; an inner row of long bracts and an outer row of lax, short bracts in *Chionoracium*, respectively).

While the richness of forms and the taxonomic complexity of the predominantly apomictic (sub)genera *Hieracium* and *Pilosella* has attracted the interest of botanists from the middle of the 19th century [3,32], subgen. *Chionoracium* remains very poorly explored, especially in South America. A molecular phylogeny of *Pilosella* based on two plastid markers (*trnT-trnL*, *matK*) and one nuclear marker (*ITS*) included a selection of 32 taxa from subgenera *Hieracium* and *Chionoracium* [25]. The *ITS*-based phylogeny showed *Pilosella* (with monotypic *Hispidella* as sister taxon) to be monophyletic while *Hieracium* and *Chionoracium* formed together a strongly supported clade. Species of both subgenera were somewhat intermingled, and species relationships remained largely unresolved due to the high similarity of the *ITS* sequences. Trees based on the plastid intergenic spacers showed two lineages of *Pilosella* (one introgressed by genus *Andryala*) while species of *Hieracium* and *Chionoracium* were not reciprocally monophyletic. The more variable marker *trnT-trnL* resolved two branches containing North or South American (SA) taxa of *Chionoracium*, respectively, but neither of these branches contained all the NA or SA species investigated. In a phylogenetic analysis of native and naturalized North American *Hieracium* species based on two plastid DNA markers (*trnT-trnF* and *petN-psbM*), Gaskin and Wilson [33] found that all 21 species of *Chionoracium* investigated formed a well-supported monophyletic group, which, however, also contained *H. canadense*, a species that is traditionally placed in subgen. *Hieracium*. A study of Krak et al. [34] showed that, based on the low-copy nuclear markers *gsh1* and *sqs*, *Chionoracium* taxa formed well-supported clades together with *Hieracium* species, and the former appeared to be derived from the latter. However, only two species of *Chionoracium* were included in that study. While taxonomic accounts of subgenus *Chinoracium* in central America (CA) and NA have been published [18,29], for SA, only few studies have continued the basic work of Zahn [7], for example [12,35,36,37], and still, the taxon’s diversity on this subcontinent remains almost completely unknown.

The only molecular phylogeny focused on *Chionoracium* available to date [33] is based on plastid DNA (ptDNA) markers. It did not include any South American taxa of *Chionoracium*, and the sampling of subgen. *Hieracium* taxa was almost completely restricted to polyploids that are native to the Old World, but also occur as invasives in North America. Therefore, a representative sampling of European native diploid species of subgen. *Hieracium* is necessary to attempt inferring the origin of subgen. *Chionoracium*. For this purpose, also South American *Chionoracium* taxa need to be included in phylogenetic analyses, and the application of nuclear markers in addition to plastid markers is required.

To elucidate the speciation processes that have taken place in *Chinoracium*, we choose a multigene approach based on highly variable and differently inherited gene regions (maternal—ptDNA, biparental—nuclear DNA) as well as on several unlinked nuclear markers (multi-copy, low-copy). Multi-copy nuclear ribosomal (nr)DNA regions are present in numerous copies and undergo intragenomic homogenization (concerted evolution) to different degree [38,39,40]. Single- or low-copy genes are thought not to be prone to concerted evolution, but more susceptible to population genetic processes [41], and their potential for phylogenetic reconstruction has to be assessed with caution as well [42]. Like with classical taxonomy, a single set of characters or one trait (comparable to a single gene region) can be misleading or else come close to the truth. Altogether, we apply seven distinct markers, five of them unlinked: plastid *trnT-trnL* and *trnV-ndhC*, nuclear ribosomal *ITS*, *ETS* and *5S-NTS*, and parts of the low-copy nuclear genes *sqs* and *gsh1* [34]. In different groups of the Hieraciinae and in various combinations, they revealed particular aspects of speciation [25,26,43,44,45,46,47]. The majority of these markers is highly variable in the study group and consists mostly of non-coding DNA (intergenic spacers, introns). Here, we apply all these markers to subgen. *Chionoracium* and use a representative selection of diploid species of subgen. *Hieracium* from Europe along with other genera of the Hieraciinae to unravel the relationships and speciation patterns.

Specifically, we address the following questions: (1) Is *Chionoracium* nested within *Hieracium* and thus should be treated as a subgenus of *Hieracium*? (2) Can its origin from European ancestral lineages be traced? (3) How did the colonization of the New World take place and when? (4) What are the species relationships within *Chionoracium*? (5) Are they in concordance with sectional classification based on morphological characters? (6) When and where did the lineages diverge in the New World? Our study is bringing many new insights into this understudied plant group that shall provide a basis for a targeted broader sampling and eventually a revision of the entire taxon.

## 2. Results

### 2.1. Phylogenetic Inference Based on ptDNA

**Figure 1:**
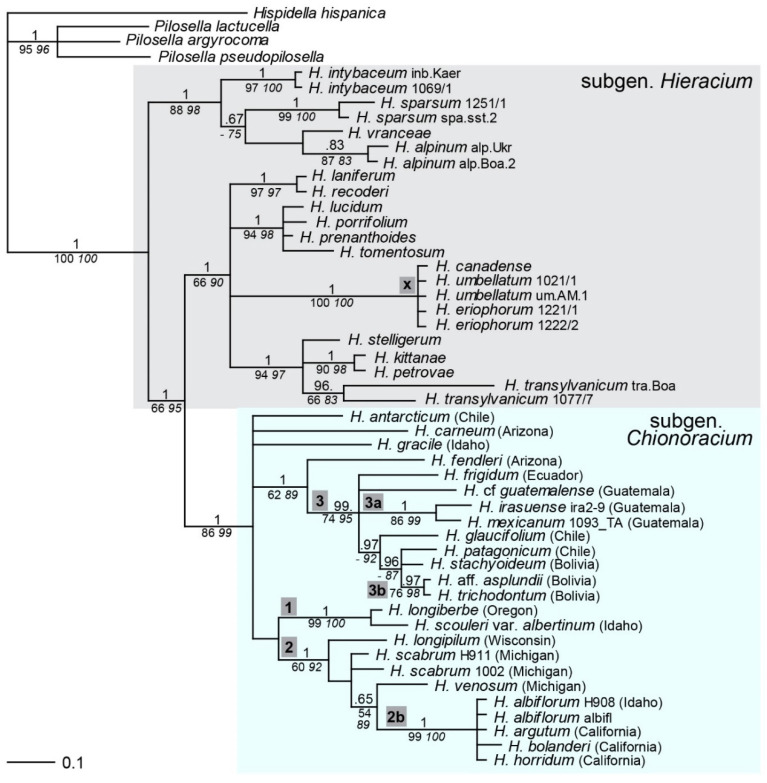
Phylogenetic analyses of subgen. *Chionoracium* based on plastid DNA (*trnT-trnL*, *trnV-ndhC*). The Bayesian consensus tree is shown with posterior probabilities (pp) above branches and bootstrap support (BS) from Maximum Parsimony (MP, regular font) and Maximum Likelihood (ML) analyses (italics) below branches. Support values are only given for branches, if pp was >0.94 or BS was >70 in at least one type of analysis. If more than one accession per species was analyzed, their labels are indicated after the species name. Numbers in grey boxes indicate monophyletic lineages mentioned in the text (see also Table 1).

Sequences of the plastid intergenic spacers *trnT-trnL* and *trnV-ndhC* were concatenated for combined analyses. All samples of subgen. *Chionoracium* formed a well-supported monophyletic clade (Figure 1) emerging from subgen. *Hieracium*. In the latter, *H. canadense* was sister to *H. eriophorum* and *H. umbellatum* (lineage x); the latter is the most widespread diploid species of subgen. *Hieracium*, which occurs in large parts of Europe, in western Asia, but also in northern NA. Within subgen. *Chionoracium*, several groups were distinguished with high support: a lineage consisting of the western NA species *H. longiberbe* and *H. scouleri* (lineage 1), a lineage comprising further seven NA species (lineage 2), among which a subset of four closely related species from western and southwestern NA (*H. albiflorum*, *H. argutum*, *H. horridum*, *H. bolanderi*) formed a derived branch (lineage 2b), and a group of ten CA and SA species (lineage 3); of these, two samples from Guatemala (*H. mexicanum*, *H. irasuense*, 3a) and two Bolivian species (*H. stachyoideum*, *H.* aff. *asplundii*, 3b) formed sister relationships, respectively. Basal to lineage 3 occurred *H. fendleri* from the southern U.S.A. Independent lineages with unclear relationships were found for *H. carneum*, *H. gracile* and *H. antarcticum*.

### 2.2. Phylogenetic Inference Based on Multi-Copy nrDNA (ITS, ETS)

*ITS* and *ETS* regions were at first analyzed separately. The *ITS* tree (Figure S1) was characterized by a basal polytomy of both *Hieracium* subgenera, from which several lineages emerged. All species of subgen. *Chionoracium* grouped together (albeit unsupported) along with one species of subgen. *Hieracium* from southeastern Europe (*H. petrovae*). Within *Chionoracium*, lineage 1 of the ptDNA tree (*H. longiberbe* and *H. scouleri*) was found again; ptDNA lineage 2 was distinguished into two well-supported groups, one comprising eastern NA species that were basal in the ptDNA tree (2a), the other group corresponded to the southwestern NA taxa derived from them (2b). Within the latter, a close relationship of *H. albiflorum* and *H. bolanderi* was found (2c). The CA + SA lineage 3 of the ptDNA tree was retrieved as well, but only one of the CA species (*H. mexicanum*) was included (3*) whereas the other two (*H.* cf. *guatemalense*, *H. irasuense*) occurred in unresolved positions. Within lineage 3*, a close relationship of the Chilenean species *H. glaucifolium* and *H. patagonicum* appeared (3c). The *ITS* tree also revealed two additional pairs of closely related species: *H. carneum*—*H. fendleri* (lineage 4) and *H. gracile*—*H. antarcticum* (lineage 5). The former samples are from Arizona (southern NA), but the latter represent extremes of the New World: Idaho and Patagonia. For a summary of lineages found with different markers, see Table 1.

The *ETS* marker showed a separation of subgen. *Hieracium* into two main clades of mainly western and eastern European origin, respectively (see also [26]). All species of subgen. *Chionoracium* emerged from the eastern European clade (Figure S2). Their common branch was unsupported, but they formed two main subclades, one consisting of exclusively NA species (comprising all species of lineages 1 and 2b), the other comprising taxa from the entire New World. Several species relationships in the latter subclade matched those retrieved previously by ptDNA and *ITS* (2a, 3*, 3c, 4, 5; Table 1). Except for NA species groups 1 and 2b, *ITS* and *ETS* trees showed the same relationships, but *ETS* provided higher resolution at the base of subgen. *Chionoracium* as well as within subgen. *Hieracium*. As with *ITS*, clade 3* of CA + SA species comprised only *H. mexicanum* whereas two other species from CA (*H.* cf. *guatemalense*, *H. irasuense*) formed independent lineages. Sister to clade 3* were *H. carneum* and *H. fendleri* (lineage 4) from southern NA.

**Figure 2:**
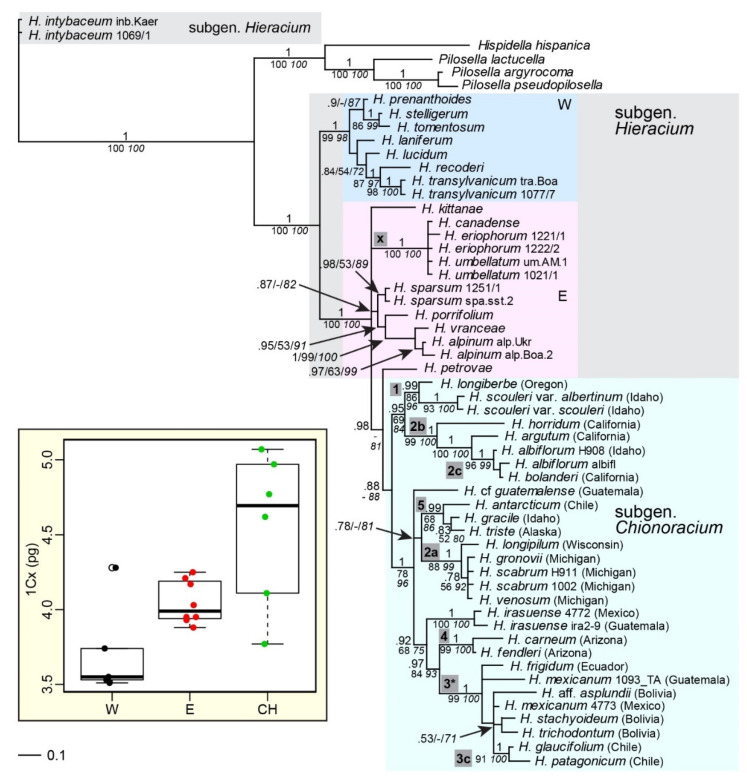
Phylogenetic analyses of subgen. *Chionoracium* based on multi-copy nrDNA (*ITS* + *ETS*). The Bayesian consensus tree is shown with posterior probabilities (pp) above branches and bootstrap support (BS) from MP (regular font) and ML analyses (italics) below branches. Support values are only given for branches, if pp was >0.94 or BS was >70 in at least one type of analysis. If more than one accession per species was analyzed, their labels are indicated after the species name. Within subgen. *Hieracium*, species groups of mainly western (W) or eastern (E) European origin are distinguished. Numbers in grey boxes refer to clades of closely related species (see Table 1). Inset: Box-plot of genome sizes of western European (W) and eastern European (E) species of subgen. *Hieracium* compared to genome sizes of subgen. *Chionoracium* (CH). The different groups differ significantly (ANOVA: F = 7.813, *p* < 0.005). For details of genome size measurements, see Table S1.

Combined analyses of concatenated *ITS* and *ETS* sequences (Figure 2) expectedly revealed the same relationships as in the individual analyses of these markers (Figures S1 and S2), sometimes with higher support. A sister relationship of subgen. *Chionoracium* with the eastern European species *H. petrovae* was found in the combined analysis.

Genome sizes of western European species of subgen. *Hieracium* are significantly lower than those of species with eastern European origin [48]. Subgen. *Chionoracium* samples were found to have genome sizes that mostly exceeded those of eastern European *Hieracia* (Figure 2, inset), largely in accordance with their placement in the tree. The only outlier was *H. albiflorum* whose genome size was markedly lower than that of other *Chionoracium* species and even lower than that of species of subgen. *Hieracium* with eastern European origin. The accession of *H. canadense* analyzed here proved to be triploid; all other species were expectedly diploid, which was occasionally confirmed by chromosome counts (Table S1).

### 2.3. Phylogenetic Inference Based on Multi-Copy nrDNA (5S-NTS)

**Figure 3:**
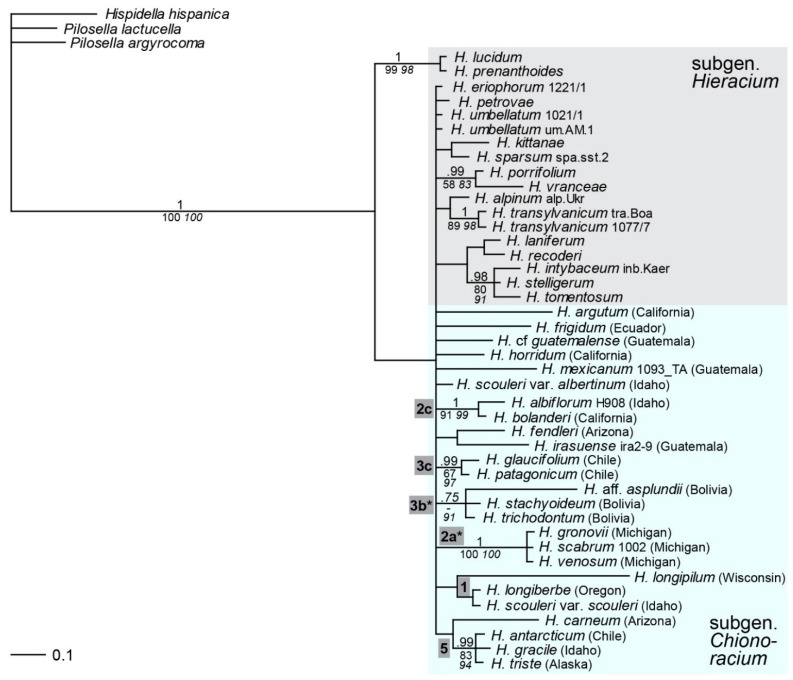
Phylogenetic analyses of subgen. *Chionoracium* based on multi-copy nrDNA (*5S-NTS*). The Bayesian consensus tree is shown with posterior probabilities (pp) above branches and bootstrap support (BS) from MP (regular font) and ML analyses (italics) below branches. Support values are only given for branches, if pp was >0.94 or BS was >70 in at least one type of analysis. If more than one accession per species was analyzed, their labels are indicated after the species name. Numbers in grey boxes refer to clades of closely related species (for explanations, see text and Table 1).

*5S* ribosomal DNA is not linked to the cistron containing *ITS* and *ETS* and evolves independently in the Hieraciinae [43] like in most other organisms [38,49]. Very little resolution of species relationships of *Hieracium* was found with the *5S* nontranscribed spacer; apart from a separation of two western European species of subgen. *Hieracium*, all other species of both subgenera formed a polytomy (Figure 3). However, a few species relationships among species of subgen. *Chionoracium* were found that matched the results of previously employed markers: the group of *H. scabrum*, *H. venosum* and *H. gronovii* (lineage 2a*, without *H. longipilum*); the group of *H. antarcticum*, *H. gracile* and *H. triste* (lineage 5); and lineages of *H. albiflorum*/*H. bolanderi* (2c) and *H. glaucifolium*/*H. patagonicum* (3c). Three Bolivian species (*H. trichodontum*, *H.* aff. *asplundii*, *H. stachyoideum*, lineage 3b*) and two western NA species (*H. longiberbe*, *H. scouleri*, lineage 1) formed poorly supported or unsupported branches.

### 2.4. Phylogenetic Inference Based on the Low-Copy Nuclear Marker Gsh1

**Figure 4:**
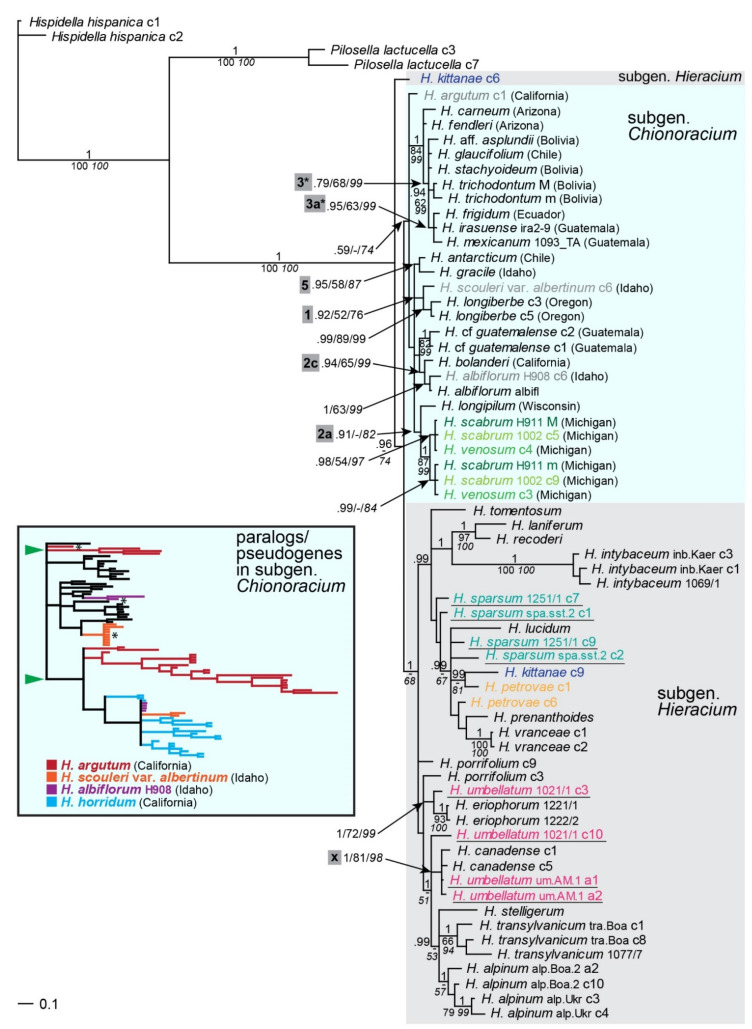
Phylogenetic analyses of subgen. *Chionoracium* based on the low-copy nuclear marker *gsh1.* The Bayesian consensus tree is shown with posterior probabilities (pp) above branches and bootstrap support (BS) from MP (regular font) and ML analyses (italics) below branches. Support values are only given for branches, if pp was >0.94 or BS was >70 in at least one type of analysis. If more than one accession per species was analyzed, their labels are indicated after the species name. Divergent alleles of the same individual are given in the same color; accessions of the same species of subgen. *Hieracium* occurring in different parts of the tree are underlined. Numbers in grey boxes refer to clades of closely related species (see Table 1). The inset shows paralogs/pseudogenes found in some North American species. For details of the analysis on which the inset is based, see Figure S3. Green arrowheads in the inset mark lineages consisting only of paralogs/pseudogenes; putative orthologous sequences are indicated by asterisks and are shown in grey in the main figure.

*Gsh1* appeared to be a single-copy gene in subgen. *Hieracium*: it was homozygous or heterozygous in diploids and never contained more alleles than chromosome sets in poly-ploids [46,47]. The *gsh1* tree (Figure 4) shows subgen. *Chionoracium* as a coherent group with one clone (allele) of the eastern European endemic species *H. kittanae* at its base; the group was paraphyletic with respect of the rest of subgen. *Hieracium*. Thirteen accessions of subgen. *Chionoracium* were homozygous, six others were heterozygous (except four in which paralogs were found, see below). Of the latter six samples, alleles of *H. trichodontum*, *H.* cf. *guatemalense* and *H. longiberbe* were coalescent. In contrast, alleles of two accessions of *H. scabrum* and one accession of *H. venosum* formed two groups, each containing one allele of each of the three accessions. In subgen. *Hieracium*, the majority of accessions was heterozygous. Furthermore, many of the alleles of this subgenus did not coalesce, or two accessions of the same species occurred in different positions with moderate or high support (*H. sparsum*, *H. umbellatum*). Both alleles of *H. canadense* clustered with both alleles of one accession of *H. umbellatum* (lineage x). Basal species relationships within subgen. *Chionoracium* remained largely unresolved, but similar groups of closely related species compared to analyses with other markers were revealed (1, 2a, 2c, 3*, 3a* and 5, see Table 1; lineages 3 and 3a showed slightly different taxon compositions). All CA and SA taxa except *H.* cf. *guatemalense* and *H. antarctium* formed a clade, to which the southern NA taxa *H. carneum* and *H. fendleri* were basal.

### 2.5. Paralogs and Pseudogenes of the Low-Copy Nuclear Marker Gsh1

In four NA accessions (*H. argutum*, *H. albiflorum* H908, *H. scouleri* var. *albertinum*, and *H. horridum*), paralogs were detected (Figure 4, inset; Figure S3). Cloned sequences of these accessions were highly divergent, exceeding by far the normal range of variation of the entire subgen. *Chionoracium*. In *H. scouleri* var. *albertinum*, two cloned sequences, very similar to each other, occurred in a very distant position to all other *Chionoracium* species whereas nine cloned sequences were identical or nearly identical to each other and grouped with *H. longiberbe*, a relationship also revealed by other markers (lineage 1). One of the latter clones (clone 6) was randomly chosen to represent the putative orthologous copy of the gene in the phylogenetic analyses. A similar pattern was observed for *H. albiflorum* H908: four nearly identical cloned sequences occurred very distantly from other *Chionoracium* species, but two similar clones clustered with the homozygous sequence of another accession of *H. albiflorum*; clone 6 had the shorter branch and was chosen to represent the sample in the phylogenetic tree. Altogether 22 clones of *H. argutum* formed three highly divergent groups outside of other *Chionoracium* species; only a single sequence (clone 1) occurred in an unresolved position among them and was included in phylogenetic analyses as a putative orthologous copy. Nineteen clones of *H. horridum* occurred in altogether four lineages; all of them were very distant from the orthologous copies of subgen. *Chionoracium*. Nested among them were also the aberrant copies of *H. albiflorum* H908 and *H. scouleri* var. *albertinum*, indicating a common origin of these variants for the three samples. *Hieracium horridum* was excluded from phylogenetic analyses with this marker, because no candidate orthologous copy was found among the clones sequenced.

Most non-orthologous clones of *H. argutum* and *H. horridum* were highly divergent at the individual level, suggesting not only a single paralogous copy (as in *H. albiflorum* H908 and *H. scouleri* var. *albertinum*), but a proliferation of duplicated (paralogous) *gsh1* sequences. Clones of these four accessions were analyzed for indications of pseudogenization; the results are summarized in Table 2. In many clones of all four accessions, an additional AG adjacent to the AG splice site of exon 13 was found; although this feature may not necessarily affect gene function, it might result in erroneous splicing. In two clones of *H. horridum* and most clones of *H. argutum*, a corrupted splice site (TG instead of AG) was found in exon 12, which is very likely an indication of pseudogenization. In six clones of *H. horridum*, a stop codon occurred in exon 13, indicating these clones are pseudogenes. In two clones of *H. scouleri* var. *albertinum* (clones 3 and 11), exon 12 and large parts of the adjacent introns were missing. The loss of an exon is very likely to render the gene nonfunctional even though it did not affect the reading frame. Another unusual feature found in the clones was an extremely long poly-T region in intron 13; it affected clones 5, 11 and 12 of *H. argutum*, which formed a separate branch in the gene tree, but also clone 19, which might be recombinant. Many clones combined two or three of these features (Table 2), but the clones of *H. albiflorum* H908 and *H. scouleri* var. *albertinum* that were chosen for phylogenetic analyses showed none of them, suggesting that they are indeed orthologous sequences. Clone 1 of *H. argutum*, used for the *gsh1*-based phylogeny, showed only the additional AG adjacent to the splice site of exon 13, but it cannot be decided if this clone is an orthologous copy or if it is recombinant between an orthologous copy (not found among the clones sequenced) and one of the paralogs; its position in the tree (Figure S3) might suggest the latter.

### 2.6. Phylogenetic Inference Based on the Low-Copy Nuclear Marker Sqs

**Figure 5:**
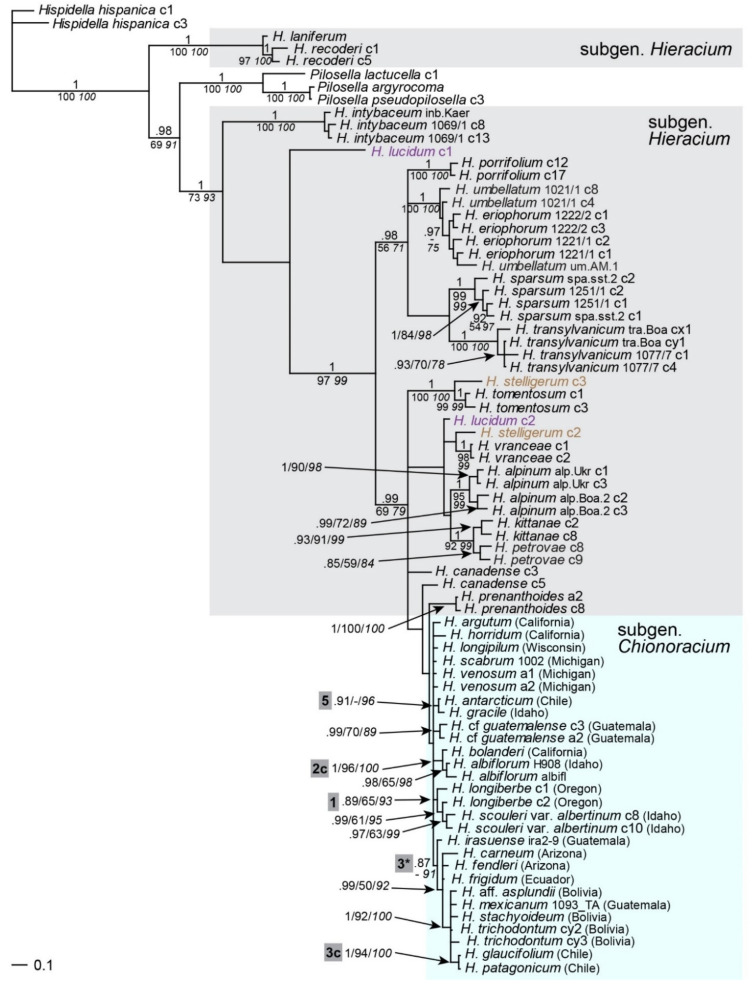
Phylogenetic analyses of subgen. *Chionoracium* based on the low-copy nuclear marker *sqs.* The Bayesian consensus tree is shown with posterior probabilities (pp) above branches and bootstrap support (BS) from MP (regular font) and ML analyses (italics) below branches. Support values are only given for branches, if pp was >0.94 or BS was >70 in at least one type of analysis. If more than one accession per species was analyzed, their labels are indicated after the species name. Divergent alleles of the same individual are given in the same color. Within subgen. *Chionoracium*, numbers in grey boxes refer to clades of closely related species (see Table 1). Clones are abbreviated with c, alleles (inferred from cloned and direct sequences) with a.

The *sqs* gene appeared to be single-copy in subgen. *Hieracium* and in the Mediterranean-Macaronesian Hieraciinae genus *Andryala* [44,45] as well as in a broad range of other Asteraceae tribes except in *Artemisia campestris* L. where two paralogous copies were found [34]. In the *sqs* tree (Figure 5), subgen. *Chionoracium* sequences were much more similar to each other than the divergence within subgen. *Hieracium*. The latter showed strongly incongruent basal species relationships, with some taxa (*H. laniferum*, *H. recoderi*) ending up in outgroup positions, and alleles of some species (*H. lucidum*, *H. stelligerum*) being very divergent from each other (for discussion, see [44]). All accessions of subgen. *Chionoracium* were found together, in a position derived from subgen. *Hieracium*, but without statistical support. Interestingly, *H. canadense* occurred near the base of this group, far apart from *H. umbellatum*, with which it formed significantly supported clusters based on all other markers (Figure 1, Figure 2, Figure 3 and Figure 4, lineage x). In contrast to *ETS* and *gsh1*, *sqs* sequences that were most similar to those of subgen. *Chionoracium* belong to a subgen. *Hieracium* species of western European origin (*H. prenanthoides*). Altogether, 18 accessions of subgen. *Chionoracium* were homozygous (in subgen. *Hieracium* only two), and even the sequences of five heterozygous individuals were highly similar to each other: two of them were coalescent (*H. scouleri* var. *albertinum*, *H.* cf. *guatemalense*), the other three (*H. venosum*, *H. longiberbe*, *H. trichodontum*) were unresolved due to the generally high *sqs* sequence similarity among most species of subgen. *Chionoracium*. Despite this low resolution, a few species relationships, corresponding to those found previously, were detected (1, 2c, 3*, 3c, 5; see Table 1).

### 2.7. Multigene Phylogeny of Subgen. Chionoracium

To possibly achieve a better resolution of species relationships of subgen. *Chionoracium*, sequences of all markers were concatenated for a multigene phylogeny. For this purpose, most species of subgen. *Hieracium* were excluded for the following reasons: (i) Strong discrepancies between the trees were found (*H. intybaceum*—ptDNA/*5S-NTS*/others; *H. recoderi*, *H. laniferum*, *H. canadense*—*sqs*/others; *H. vranceae*, *H. transylvanicum* and *H. prenanthoides*—*5S-NTS*/others; *H. sparsum*, *H. alpinum*—ptDNA/others). (ii) Some accessions showed strongly divergent alleles ending up in different parts of the tree (*H. lucidum* and *H. stelligerum*—*sqs*; *H. kittanae*—*gsh1*). (iii) Accessions of the same species were not monophyletic, but occurred in different lineages (*H. umbellatum* and *H. sparsum*—*gsh1*). *Sqs* and *gsh1* sequences of the remaining heterozygous samples were very similar and were therefore represented as single sequences using IUPAC ambiguity codes to account for substitutions between them. For subgen. *Chionoracium*, the *gsh1* sequence of *H. argutum* was substituted by missing data, because its identity as an orthologous copy remained dubious.

**Figure 6:**
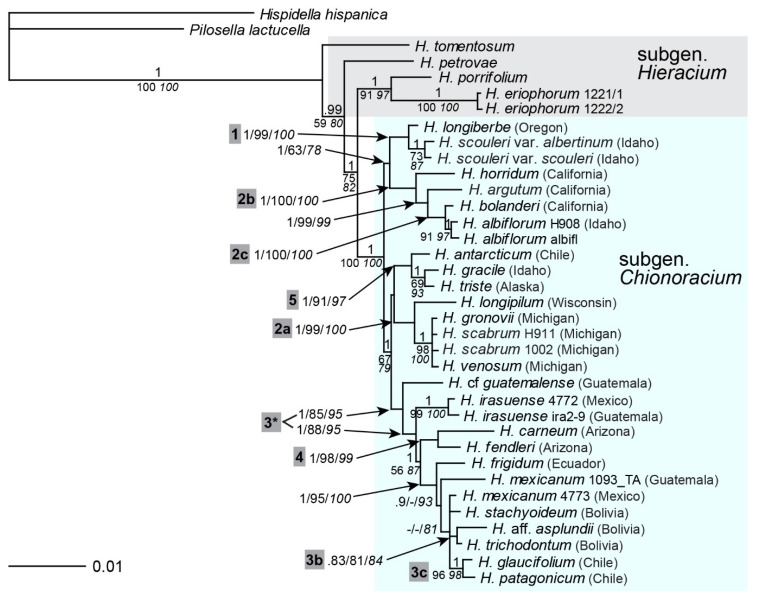
Multigene phylogenetic analyses of subgen. *Chionoracium.* The Bayesian consensus tree is shown with posterior probabilities (pp) above branches and bootstrap support (BS) from MP (regular font) and ML analyses (italics) below branches. Support values are only given for branches, if pp was >0.94 or BS was >70 in at least one type of analysis. If more than one accession per species was analyzed, their labels are indicated after the species name. Grey boxes with numbers indicate lineages of closely related species. 3*—small alterations of the taxon composition of lineage 3.

Subgenus *Chionoracium* formed a strongly supported branch derived from subgen. *Hieracium*; species of eastern European origin (*H. petrovae*, *H. porrifolium*, *H. eriophorum*) showed sister relationships to *Chionoracium* (Figure 6). All species relationships indicated by several markers were expectedly retrieved (Table 1). The lineage comprising all NA species (2) and the close relationship of *H. mexicanum* and *H. irasuense* (lineage 3a) inferred only from ptDNA did not occur in the combined dataset, because all nuclear markers suggested the presence of 2–3 NA lineages. Among the latter, lineages 1 and 2b comprising western and southwestern NA species formed a sister relationship whereas lineage 2a composed of eastern NA taxa occurred in a more distant position. Except for *H. antarcticum*, all CA and SA taxa formed a well supported lineage (3*). Nested within this lineage were *H. carneum* and *H. fendleri* from southern NA (lineage 4); they are sister to the CA and SA species. Of the three CA species, only *H. mexicanum* clustered with the SA taxa, two others (*H.* cf. *guatemalense*, *H. irasuense*) occupied basal positions.

### 2.8. Sectional Classification in Comparison with Phylogenetic Reconstruction

Generally, the traditional sectional classification of *Chionoracium* (Table S2) did not correspond well to the results of the phylogenetic analyses. Species traditionally placed into sections *Aurelliformia*, *Cynoglossoidea*, and *Stenotheca*, respectively, did not form mono-phyletic groups. Section *Aurelliformia* consisted of all species of lineage 5 (nNA and sSA, disjunct distribution), but it also contained two species of lineage 3 that comprises all other South American species, which are genetically very similar. Besides, SA species of sections *Mandonia* and *Piloselliformia*, represented here by one and two species, respectively, also belonged to lineage 3. Furthermore, the assignment of *H*. cf. *guatemalense* to sect. *Aurelliformia* proposed by Standley and Steyermark [50] did not match our results, because this Central American species usually occurred in an isolated position in the trees and was not directly involved in the lineage that gave rise to the South American radiation. Furthermore, sections *Cynoglossoidea* and *Stenotheca* comprise species from western as well as eastern North America that formed separate phylogenetic lineages with all nuclear markers.

On the other hand, our results showed close relationships between *H*. *carneum* and *H*. *fendleri* (lineage 4), traditionally placed in section *Crepidispermum*. Our data also revealed close relationships between *H*. *albiflorum* (sect. *Stenotheca* = sect. *Pulmonareiformia* in Zahn [7]) and *H*. *bolanderi* (sect. *Cynoglossoidea* = sect. *Thyrsoidea* in Zahn [7]) as suggested by Beaman [29] based on morphological characters.

### 2.9. Dating of Chionoracium Lineages

We conducted a Bayesian divergence time estimation based on *ITS*/*ETS* incorporating three fossil constraints. To evaluate the statistical support in our analytical framework, we first ranked the calibration schemes according to their marginal likelihood estimates from the nested sampling analyses (Table S3). To obtain the Bayes factors, we then computed for each scheme the marginal log likelihood difference with the top performing scheme. All the worst performing schemes included a STRICT clock model. The fit of the best scheme compared to the ones with a STRICT clock lent overwhelming support to the former (BF > 39), which is evidence for a non-negligible amount of molecular rate heterogeneity among lineages that cannot be handled with a constant rate model. Bayes factors comparison strongly supported a calibration scheme that associates the optimized relaxed clock parametrized with a lognormal distribution (ORC-LN) model with the birth-death process and the GTR + Γ4 + I model, which was subsequently applied to date the lineages. For an overview of dating results, see Table 3.

**Figure 7:**
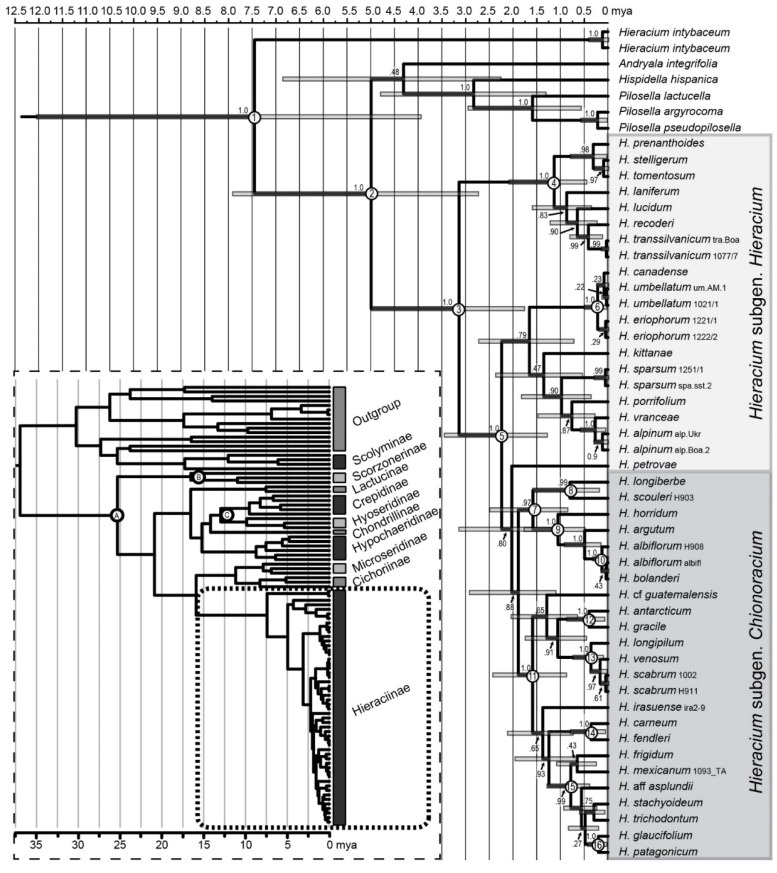
Chronogram of Hieraciinae. Nodes are drawn at their posterior median age in millions of years ago (mya) estimated by BEAST under the optimized clock model, a birth-death tree prior and using three calibrated points. The inset figure shows the whole timetree wherein the fossil constraints are indicated (A: *Cichorium*-type pollen, B: *Scorzonera*-type pollen, C: *Sonchus*-type pollen). Numbers associated with nodes designate the divergence events whose dates are reported in Table 3. Horizontal bars represent the 95% posterior consistency index for the node ages, posterior probabilities of nodes are indicated.

The common node of *Hieracium* subgenera *Hieracium* and *Chionoracium* was dated at 3.14 million years ago (mya) (Figure 7, node 3), and subgen. *Chionoracium* diverged from the eastern European lineage of subgen. *Hieracium* around 2.24 mya (node 5). Most of the western North American *Chionoracium* species had a common ancestor (node 7) whereas the rest of the subgenus formed a separate lineage (node 11); both diverged almost simultaneously 1.58 and 1.59 mya, respectively. Among the western NA species, lineage 1 was dated at 0.82 mya (node 8) and lineage 2b at 1.05 mya (node 9). Within the latter, the very closely related species *H. albiflorum* and *H. bolanderi* (lineage 2c) split only about 40,000 years ago (node 10). The common clade of eastern NA taxa (lineage 2a) dated back to 0.36 mya (node 13), similarly like lineage 5 comprising *H. gracile* (northern and western NA) and *H. antarcticum* (southern SA) that split 0.4 mya (node 12). Furthermore, the (south)western NA species *H. carneum* and *H*. *fendleri* (lineage 4, section *Crepidispermum*) diverged around the same time (0.36 mya, node 14). The clade of all South American species (except *H. antarcticum*) had a common ancestor 0.79 mya (node 15) with the Central American species *H. mexicanum* nested among them. The southern SA lineage, represented by *H. glaucifolium* and *H. patagonicum* (lineage 3c), diverged from the latter about 0.2 mya (node 16). Around the same time, *H. canadense* (subgen. *Hieracium*) diverged from the European clade involving *H. umbellatum* (0.22 mya, node 6).

## 3. Discussion

### 3.1. Features of the Molecular Markers

For a proper interpretation of the phylogenetic patterns, it is important to understand the propensities of the molecular markers applied here. Furthermore, the variability of the individual markers differed and so did their potential to unravel species relationships.

The plastid DNA markers showed the lowest proportion of parsimony informative (PI) sites, even though combined they had the highest number of total characters. This lower variation of ptDNA compared to nuclear markers is typical for plants [51]. Nevertheless, ptDNA showed a good resolution of *Chionoracium* species relationships. Generally, it found more inclusive groups of NA and CA + SA species than nuclear markers as well as close relationships of two species pairs from Guatemala and Bolivia, respectively, that were not evident with nuclear markers. At the same time, it failed to reveal several groups of closely related species found with most other markers. However, the discrepancies between ptDNA and nuclear DNA markers were largely due to a lack of resolution in different parts of the tree rather than due to strong conflict that might be attributed to chloroplast capture.

The multicopy nuclear markers *ITS*, *ETS* and *5S-NTS* were generally well homogenized. *ITS* and *ETS* were about four times as variable as ptDNA, and *5S-NTS* was about seven times as variable. The latter showed the highest proportion of PI characters of all datasets, but it had the shortest sequence. The propensity of the nrDNA markers to resolve species relationships different drastically. While *ITS* and *ETS*, individually and combined, showed a fair resolution of *Chionoracium* relationships, *5S-NTS* revealed only a few crown group relationships even though the number of PI characters was not much lower than that of *ITS* or *ETS*. This pattern has been described before in subgen. *Hieracium* [43], but it contrasts with many plant groups where not only higher sequence variation of *5S-NTS* over *ITS* or *ETS*, but also more powerful resolution of species relationships was found (e.g., Malvaceae [52], Potamogetonaceae [53], Poaceae [54]). The reasons for this unusual behavior in *Hieracium* are unknown, but apparently it also applies to subgenus *Chionoracium*.

The low-copy nuclear markers *gsh1* and *sqs* were specifically developed with the purpose to obtain highly variable markers for resolving close interspecific relationships in the Hieraciinae and other Asteraceae [34]. Both contain several highly variable introns, and the sequences were about five times as variable as ptDNA; also, the number of PI characters of each dataset was higher than that of *ITS* and *ETS* combined. Nevertheless, the overall diversity within subgen. *Chionoracium* was rather low compared to that within subgen. *Hieracium*. Consequently, only a moderate number of well supported species relationships in *Chionoracium* was found. Furthermore, allelic divergence of heterozygous plants was much lower in *Chionoracium*, which also showed a much higher degree of homozygous individuals than subgen. *Hieracium*. In the latter and in the closely related genus *Andryala*, species relationships were confounded by large allelic divergence in *sqs* [44,45]. However, in *Andryala*, the marker revealed founder events of endemic Macaronesian species [45] confirming that population bottlenecks related to the colonization of new habitats can lead to a loss of genetic diversity and/or locus loss of low-copy nuclear markers [41]. This also appears to be the case in *Chionoracium*, because the overall similarity of *sqs* sequences in this group—despite the otherwise high level of variation of this marker in other Hieraciinae—could point, like ptDNA, to a single colonization of the New World.

### 3.2. Paralogs and Pseudogenization of Gsh1 in Some Samples of Chionoracium

In four accessions, highly divergent *gsh1* paralogs were found, many of which showed indications of pseudogenization. These samples are from western and southwestern NA (Idaho, California), and the gene tree as well as the geographic origins suggest that the gene duplication may have been a single event, followed by different fates and further proliferation of the paralogs in these accessions. However, while three of them (*H. horridum, H. argutum,* and *H. albiflorum*) are closely related based on several markers (lineage 2b), the fourth (*H. scouleri* var. *albertinum*) is sister to *H. longiberbe* (lineage 1). Only in the ptDNA tree, all four species form a clade (lineage 2). Furthermore, other accessions of *H. albiflorum* and *H. scouleri* did not show the duplication, and neither did the closely related species *H. bolanderi*. A possible explanation in keeping with the patterns is that the gene duplication has indeed occurred before these species diverged and that the paralogs were subsequently purged from most genomes of the species affected. Alternatively, the gene duplications may have occurred independently, but we consider this as less likely because of the high sequence similarities and the aberrant features of the paralogous clones shared by several accessions. Paralogs in *gsh1* have been detected here for the first time; the gene appeared to be single-copy not only in subgen. *Hieracium* [46,47], but also in a broad range of other Asteraceae tribes [34].

### 3.3. Maternal Lineages of Chionoracium

The phylogeny of maternal lineages based on ptDNA showed subgen. *Chionoracium* as a monophyletic group in accordance with the results of Gaskin and Wilson [33] that were based on partly different ptDNA regions. In our tree, *Chionoracium* occurred in a position derived from subgen. *Hieracium* whereas in the former study, species of subgen. *Hieracium* occupied unresolved positions basal to the lineage of *Chionoracium*. This discrepancy is most likely due to the use of different outgroups and a different taxon sampling of subgen. *Hieracium*. While Gaskin and Wilson [33] focused on species of subgen. *Hieracium* that are invasive in North America, our sampling of this subgenus is representative with respect to diploids and covers the main evolutionary lineages [26,43,44]. Gaskin and Wilson [33] also showed that *Crepis* is not closely related to *Hieracium*, refuting the classification of Sell [13].

Most *Chionoracium* species relationships we found expectedly corresponded to those in Gaskin and Wilson [33] who, however, did not investigate any South American taxa. A striking discrepancy between the two ptDNA phylogenies concerns the position of *H. canadense*, which clustered with *Chionoracium* species from western NA (our lineage 2b) in the tree of Gaskin and Wilson [33], but with *H. umbellatum* in our study (lineage x, Figure 1, Figure 2, Figure 4), to which it is very similar morphologically, and it has even been synonymized with *H. umbellatum* [18]. Gaskin and Wilson [33] investigated two samples of *H. canadense*, which showed the same maternal origin and attributed its placement in the ‘wrong’ subgenus to chloroplast capture. The sample analyzed here was triploid (Table S1), and in the *sqs* tree (Figure 5), it occurred near the base of subgen. *Chionoracium*, very distant from *H. umbellatum*. We therefore assume an allopolyploid origin of our sample of *H. canadense*. It is possible that those plants of unknown ploidy investigated by Gaskin and Wilson [33] may be allopolyploids as well, in which case the putative hybridization between the subgenera has occurred in opposite directions with respect to the maternal parent.

Within subgen. *Chionoracium*, the largest clade of NA species (lineage 2) showed eastern NA taxa basal to a derived clade of very closely related western NA species (lineage 2b), which may indicate colonization in this group from east to west, given that ptDNA is known to reflect geographic patterns [55,56,57]. On the other hand, the derived position of lineage 2b is only significant in ML analyses (Figure 1), and its long branch compared to those of other species in lineage 2 indicates different rates of molecular evolution which might have caused difficulties in phylogenetic analyses. Interestingly, another lineage of western NA species (lineage 1) formed a separate branch emerging from the basal polytomy of subgen. *Chionoracium* despite the partly overlapping distribution areas. Geographic patterns were also evident concerning SA species. All of them (except *H. antarcticum*, see below) were very closely related and occurred in the most derived positions, indicative of a rapid speciation in the Andes. Basal to them (lineage 3) occur all CA species, and sister to all of them is *H. fendleri* from the southern U.S.A., which is in keeping with a gradual colonization of South America from the north.

### 3.4. Evidence from Nuclear Markers and Genome Size

All nuclear markers (except *5S-NTS* with the lowest resolution) showed subgen. *Chionoracium* as a group, however, with none of them, it obtained statistical support. In the *gsh1* tree, the subgenus was most clearly distinguished statistically, but it nested between one allele of the eastern European species *H. kittanae* and the rest of subgen. *Hieracium*. With all other nuclear markers (as well as ptDNA), subgen. *Chionoracium* was derived from subgen. *Hieracium*. Furthermore, according to *ITS*, *ETS* and *gsh1*, various eastern European species were most closely related to subgen. *Chionoracium.* In contrast, the most similar *sqs* sequences belonged to a western European species (*H. prenanthoides*). This species has ancient hybrid origin combining eastern as well as western European lineages [26,47]. If the ‘western’ *sqs* allele was lost in this species and only the ‘eastern’ allele was maintained, the apparent contradiction with other nuclear markers disappears.

*ETS* showed a strongly supported clade comprising subgen. *Hieracium* species of eastern European origin along with subgen. *Chionoracium* species. The *ETS* region is the best approximation of species relationships in subgen. *Hieracium* as it is in accordance with geographic origins and genome size [43,44]. *Hieracium* species of western European origin have significantly lower genome sizes than those of eastern European origin [48]. In keeping with the *ETS* phylogeny, genome sizes of most *Chionoracium* samples matched or exceeded those of eastern European *Hieracium* species. This is strong additional evidence for an eastern European origin of the ancestors of subgen. *Chionoracium*.

### 3.5. Synthesis of Phylogenetic Analyses

All molecular markers unambiguously confirm *Chionoracium* as a subgenus of *Hieracium*. Only with ptDNA, *Chionoracium* formed a well-supported monophyletic group. Because the effective population size of a haploid (plastid) genome is twice smaller than that of a diploid (nuclear) genome in monoecious plants [58], coalescence times of ptDNA haplotypes are shorter. Apparently, the time for nuclear genes to coalesce was still insufficient. Consequently, all markers revealed unresolved basal relationships among *Chionoracium* indicated by polytomies in the trees. With *ETS*, the marker providing almost completely resolved relationships of *Chionoracium* (Figure S2), the basal polytomy concerned the entire eastern European clade of *Hieracium*. This can be interpreted as incomplete lineage sorting caused by ancient rapid radiations, occurring at the level of the origin of subgen. *Chionoracium* as well as in the initial phases of its radiation in the New World. A further, much more recent case of incomplete lineage sorting (or simply a lack of genetic diversification) is evident with the South American lineage.

All molecular markers resolved groups of closely related species. Partly contradictory relationships found with different markers concern the number and arrangement of North American lineages as well as the position of southern NA and CA species relative to each other and to SA taxa. However, a sister relationship of *H. carneum* and *H. fendleri* from southwestern NA to the majority of CA and SA species is evident with most nuclear markers, suggesting a gradual colonization from north to south. The CA species most closely related to SA species is *H. mexicanum* whereas *H.* cf. *guatemalense* appeared as rather distantly related with most markers, and the position of *H. irasuense* varied with respect to southern NA and SA taxa. Central American species were never monophyletic. We assume that CA was colonized from NA several times and that apparently only the lineage of *H. mexicanum* gave rise to the South American diversity.

### 3.6. Morphological Classification in the Light of Phylogenetic Reconstruction

Some stark incongruences of molecular phylogeny with morphologically defined sections (Table S2) in the subgenus *Chionoracium* are evident: (1) Some species are merged into the same section whose lineages arose independently. For example, sections *Cynoglossoidea* and *Stenotheca* comprise species from western as well as eastern North America that formed separate phylogenetic lineages; also, species classified in sect. *Aurelliformia* belong to unrelated clades. These discrepancies can be best explained as parallel evolution of morphological traits and/or a retention of predominantly plesiomorphic characters. (2) Genetically very closely related species are split into several sections. For example, South American species are classified into sections *Aurelliformia*, *Mandonia* and *Piloselliformia*. Here, some distinct morphological diversification is evident that was probably caused by a rapid radiation in the highly diverse Andean habitats and that was not accompanied (yet) by marked genetic diversification.

Only a few cases were found when genetic and morphological evidence showed the same result: all concern pairs of very closely related species (lineages 2c, 4, and 5). Generally, the sectional classification of subgen. *Chionoracium* requires a thorough revision, as the last comprehensive treatment was published hundred years ago [7], and only a few corrections and additions were proposed since then.

### 3.7. Dating of Hieraciinae and Chionoracium Nodes

Our dating analysis was based on combined *ITS*/*ETS* markers and comprised species of many Asteraceae tribes as outgroup as well as a representative sampling of species of tribe Cichoreae and subtribe Hieraciinae. Bayes factor comparison revealed that the best fitting combination associated the ORC-LN model with the birth-death process and the GTR + Γ4 + I model. The clear rejection of the STRICK clock branch rate model is testament to the presence of substantial rate heterogeneity among lineages. Such heterogeneity could be explained by biological causes such as variation in life-history traits, generation time, population size, niche structure, or exposure to mutagens [59,60].

Within the tribe Cichorieae, we recovered similar dates to Tremetsberger et al. [61] for the major divergence events, which is unsurprising since our estimation relied on the same marker, dating method, branch rate model and calibrated nodes. Interestingly, the top five likelihood models included different combinations of branch rate and tree branching models. Yet, they were all contained within ten points of Ln(BF), but their age estimates were significantly different for the node of subtribe Hieraciinae. This indicates that our divergence times are not robust to variation in models, parameters, and priors. We speculate that this instability together with the rather large 95% highest posterior density (HPD) intervals in our focal clade could be ascribed to the joint influence of poorly resolved topology and the availability of only a single relevant calibration point deep in the tree. The rejection of the STRICT clock implies that the rates inferred in one part of the phylogeny serve as a poor proxy for estimating divergence times in other clades [62], which means that the deepest calibrated *Cichorium*-type node is the only time calibration that can directly impact the dating of the Hieraciinae node since the two other calibration nodes are not ancestral to it. Therefore, the precision of the dating hinges on the proper estimation of the branch length leading from the *Cichorium*-type node to the Hieraciinae node. However, given the shortness of our alignment, it is plausible that the model cannot properly correct for multiple substitutions and thus is unable to accurately estimate the relevant branch length. The extremely fragmentary fossil record in plants makes it difficult to reduce the uncertainty of divergence times for nodes that are not directly subtending the calibration points. Even if genome size data were used to ideally estimate sequence distances and branch lengths without random errors, the uncertainty in the temporal placement of fossils will remain a major influence on the posterior time estimates [63]. We argue that an additional source of error for the temporal dynamics inference in the Hieraciinae comes from the problematic estimation of the diversification parameters of the branching model. In the BEAST framework, the age of calibrated nodes is constrained by user specified density distribution whereas the non-calibrated nodes have prior densities specified by the branching model (the birth-death or the Yule process). This means that the influence of the branching process increases with the phylogenetic distance between the dated clade and the calibrated nodes. This influence would be particularly strong in our focal clade where all dating information is derived from a single calibration density at 22 mya. The phylogenetic patterns in our study are interpreted below as characteristic attributes of rapid evolutionary radiations. Furthermore, the intensity of taxon sampling is not uniform, with a much more densely sampled Hieraciinae clade compared to the other parts of the Cichorieae tree. Taken together, these elements suggest that there may be significant differences in diversification parameters among clades, which violates a central assumption of Bayesian dating that uses constant diversification priors for the tree branching process [64]. Given these inevitable limitations of the analysis, we will interpret the dating results with appropriate caution.

The common node of eastern European species of subgen. *Hieracium* and all species of subgen. *Chionoracium* was dated at 2.24 (1.29–3.47) mya. The node comprising all *Chionoracia* was unsupported (Figure 7) and therefore will not be considered here, but the two most basal nodes of subgen. *Chionoracium* (7 and 11) were dated at 1.58 (1.19–3.16) and 1.59 (0.89–2.45) mya, respectively. Thus, the divergence of the native American hawkweeds from European ancestors occurred in a range of 1.58–2.24 (0.89–3.47) mya, i.e., during the Pleistocene; if the error ranges are considered, the earliest divergence estimate of *Chionoracium* lies in the late Pliocene. One of these nodes (7) comprised only species from western North America and gave rise to two clades (nodes 8 and 9) dated at 0.82 (0.20–1.61) and 1.05 (0.50–1.79) mya, respectively. These are the oldest lineages within *Chionoracium*. Basal node 11 also comprised North American species that are unrelated and distributed in western, northern, and eastern NA (Table S2), but their divergences are by far younger (0.36–0.4 mya, nodes 12–14). The lineage that gave rise to the entire South American diversity (node 15) was dated at 0.79 (0.40–1.31) mya (mid Pleistocene). Thus, the colonization of the Andes via Central America took place after the closure of the Isthmus of Panama dated at ca. 3 mya [65] as well as after the final orogeny of the Andes about 2–6 mya [66,67] and is therefore independent of these geological events. This contrasts with most diversification patterns found in other plant groups [68,69] or in animals [70,71] where bursts of speciation are related to the uplift of the Andes. The Patagonian lineage of *Chionoracium* (node 16) is derived from the Andean species and diversified as late as 0.2 (0.04–0.45) mya. Even though our sampling of South American species is poor compared to the total species number (>100), the oldest lineages of *Chionoracium* belong to NA species (node 7). In addition, two Central American species (*H.* cf. *guatemalensis*, *H. irasuense*) whose origin cannot be dated due to a lack of node support are the basalmost lineages of all other *Chionoracia* (node 11). The clade of South American species includes *H. mexicanum*, revealing the Central American lineage from which the Andes were colonized. We therefore envisage that further sampling of South American hawkweeds will only confirm our results and that attempting to resolve their species relationships would require different approaches, because even the most variable molecular markers we applied here hardly showed any genetic differences of Andean species, which resulted in polytomies in all phylogenetic trees. Further examples of Andean clades that remain largely or completely unresolved concern Bromeliaceae [72] and Lamiaceae [73].

### 3.8. Colonization of the New World

Two scenarios for the colonization of the New World are possible: (1) westwards via the North Atlantic land bridge, or (2) through Asia via Beringia.

Regarding (1), while it is somewhat counterintuitive to assume a westward migration of species with eastern European origin, there are several indications that this may indeed have happened. (i) *Hieracium umbellatum* has eastern European origin, but it is the most widespread diploid *Hieracium* and occurs in large parts of Europe. In the New World, it is currently distributed over the northern and central part of NA, southwards to Oregon, Colorado, Missouri, and North Carolina, and it gave rise to *H. canadense*. (ii) Not only *H. umbellatum*, but also triploids of *H. alpinum*, another species of eastern European origin, are found as far west as Iceland and Greenland and may represent remnants of a migration via the North Atlantic land bridge (habitats there are not suitable nowadays for other species). It was supposed that migration via the North Atlantic land bridge was relatively continuous during the late Cretaceous and Paleogene (up to the early Eocene) with warm and moist climate. However, recent studies (mostly based on fossils from Iceland) indicate that migration via this land bridge was possible for temperate plant species until the latest Miocene [74,75]. This is about twice as old as the divergence of subgen. *Chionoracium* from *Hieracium* and therefore, colonization of the New World via this route is rather unlikely.

Regarding (2), there are some indications that support a colonization of the New World via Beringia. (i) *Hieracium umbellatum* is widespread throughout the Russian Far East. (ii) Western North American species form the oldest lineages in *Chionoracium* (nodes 8 and 9). The Beringian intercontinental land connection was exposed during the Tertiary and then flooded 4.8–5.5 mya [76]. In the Pleistocene, the sea level changed, exposing and flooding the region repeatedly. During the ice ages, it was not glaciated and was covered by a grassland steppe [77,78]. Therefore, colonization via this route is in much better agreement with an initial divergence of *Chionoracium* from subgen. *Hieracium* in eastern Europe about 1.59–2.24 mya, especially as the actual colonization of the New World must be younger still.

### 3.9. A Case of Long Distance Dispersal

While most *Chionoracium* species are related to species with similar geographic distribution, *H. antarcticum* from southern Patagonia was most closely related with *H. gracile* and *H. triste*, two species from western and northwestern NA. Their close relationship was supported by all nuclear markers (for *H. triste*, only sequences of *ETS* and *5S-NTS* were available). Interestingly, synonyms of *H. antarcticum* list it as a subspecies of *H. gracile* (*H. g.* ssp. *antarcticum*, *H. g.* ssp. *andinum*, *H. g.* ssp. *myosotidifolium*). Recently, *H. gracile* was synonymized with *H. triste* [18]. Thus, the close relationship between these species was already suggested on morphological grounds. However, while *H. gracile* and *H. triste* may be indeed conspecific due to the high sequence similarities, Patagonian *H. antarcticum* is most likely a different species as sequences of all markers are different from the NA species and are in the range of interspecific differences in other closely related *Chionoracia*.

The disjunct distribution of *H. antarcticum* and H. *gracile*/*H. triste* can be best explained by long distance dispersal mediated by migratory birds, which has been described or inferred for various terrestrial and aquatic plants [79,80,81,82]. There exist three flyways of migratory birds of the order Charadriiformes that range from the high latitudes of North America to Patagonia [83]. More than 20 vascular plant species from 18 genera and 10 families are known whose populations occur discontinuously in the polar regions of Arctic and Antarctic hemispheres, but bipolar distributions also occur at higher taxonomic rank [81] as in our case.

Alternatively, the splitting of the continuous geographical range of an ancestral taxon into two or more parts by the development of barriers to dispersal and gene flow (such as mountain uplift or the opening of an ocean basin; vicariance; [81]) or convergent (parallel) evolution [84] can be suggested. However, such barriers did not develop in the American mountain chain in the Pleistocene (the age of the node is ca 0.4 mya) and climatic-driven extinction reducing the former continuous range to the extreme disjunction observed today is little plausible. Parallel evolution can be excluded as well as the species form a monophyletic group.

### 3.10. Establishment of Species and Reproductive Assurance

Colonization of new areas might be facilitated by self-fertilization or by apomixis. Although reproductive modes in subgen. *Chinoracium* are still poorly known, it has been shown that both outcrossing and selfing species/populations occur in this taxon. Guppy [85] proved a high seed set after self-pollination in *H*. *albiflorum* and *H*. *scouleri*, which may, besides other factors, explain their large distribution ranges. For *H. albiflorum*, its exceptionally low genome size compared to other *Chionoracia* may additionally have favoured broader dispersal due to faster growth rates [86,87]. Self-compatibility was also observed in *H*. *abscissum*, *H*. *mexicanum*, *H*. *pringlei* and *H*. *gronovii* [29]. In *H*. *venosum* and *H*. *scouleri*, outcrossing strongly prevails [85], but also these species can produce a small portion of seeds via self-fertilization.

Reproductive modes in subgen. *Chionoracium* are in stark contrast to those in subgen. *Hieracium*, where the few extant diploids are strictly outcrossing unless pollen of another species causes a breakdown of self-incompatibility [88] and where apomixis is the dominant reproductive mode [16]. In subgen. *Chinoracium*, apomixis was not reliably confirmed and is also not very likely to occur if, as far as known, the species are diploid. Thus, the two subgenera developed different strategies of reproductive assurance that facilitated the colonization of new areas. In subgen. *Hieracium*, the formation of unreduced gametes, a prerequisite for apomixis, appears to be related to climatic changes during the Pleistocene, which enabled apomictic plants to quickly colonize deglaciated areas after the retreat of glaciers whereas diploids are nowadays mostly confined to glacial refugia, and many of them are rare or endangered [26]. In subgen. *Chionoracium*, which arose from subgen. *Hieracium* at a time when the genus was still entirely diploid and apomixis was not yet developed, the colonization of new habitats in the New World largely relied on selfing for reproductive assurance and may have particularly facilitated the generation of the large species numbers of *Chionoracia* in the Andes.

## 4. Materials and Methods

### 4.1. Plant Material

Some Central and South American samples of subgen. *Chionoracium* were collected in the field; the majority of SA material was isolated from herbarium specimens. Seeds of two NA samples were obtained from a company, seeds of further NA taxa were provided by L.M. Wilson (Abbotsford, British Columbia, Canada), and isolated DNA of some NA species was provided by J.F. Gaskin (Sydney MT, USA). Altogether, 30 samples of 25 species with a distribution from Alaska to Patagonia are included (Appendix A). For subgen. *Hieracium*, a selection of diploid European species representing the major evolutionary lineages [43] was chosen plus an accession of *H. canadense* different from the one used by Gaskin and Wilson [33]. Based on our previous results [26,44], *Hispidella hispanica* and some species of the sister genus *Pilosella* were used as outgroup. In total, we investigated 57 accessions of 46 species. Details about the samples’ origins and voucher information are provided in Table S4.

### 4.2. Genome Size Determination

Genome size of *Chionoracium* plants for which suitable fresh material was available was determined by flow cytometry using a Partec CyFlow cytometer (Partec GmbH, Münster, Germany) equipped with a green solid-state laser (Cobolt Samba, 532 nm, 100 mW output power). *Zea mays* ‘CE–777′ (2C = 5.48 pg [89]) was used as an internal standard. The modified two step-procedure described by Otto [90] was employed for sample preparation as described in [48]. Usually, 5000 nuclei were analysed for each sample. Nuclear genome size was calculated as a linear relationship between the ratio of 2C peaks of sample and standard. Individual plants were analysed three times on three different days to minimize between-day fluctuations of the instrument, and the arithmetic mean was taken as the result. If the values between the days varied excessively (by more than 2%), another analysis was conducted, and its result replaced the outlier value. The coefficients of variation (CVs) of G_0_/G_1_ peaks did not exceed 5%. Results of the measurements are provided in Table S1. Genome sizes for subgen. *Hieracium* were adopted from Chrtek et al. [48] and Mráz et al. [46].

### 4.3. Molecular Procedures

DNA was isolated from fresh, CTAB-conserved or herbarium material using a sorbitol extraction procedure [91]. PCR amplification of the markers, purification of the products and Sanger sequencing were done as described previously: *trnT-trnL* and *ITS* [25], *trnV-ndhC* and *sqs* [34,44], *ETS* [26], *5S-NTS* [92], *gsh1* [46]. Sequencing was done in both directions, and for *trnV-ndhC*, *sqs*, and *gsh1*, also internal primers were sometimes employed to obtain full reads and information about polymorphisms in nrDNA and heterozygous low-copy nuclear markers as described in the respective studies cited above. If direct sequences of nuclear markers were not homogeneous, PCR products were cloned as described in [26]. Several clones per accession were sequenced and compared with polymorphic direct sequences to ensure that all copy types were found; recombinant clones were discarded, and polymerase errors corrected as described in [44]. Sequences were submitted to GenBank (accession numbers MZ322103–MZ322303, MZ329404–MZ32942, see also Appendix A) and aligned by hand in BioEdit v7.3 (BioEdit, RRID:SCR_007361) [93]. Features of the datasets (number of characters and indels, number and proportion of parsimony informative characters) are summarized in Table S5.

### 4.4. Phylogenetic Analyses

Indels for all datasets were coded with FastGap v1.2 (FastGap, RRID:SCR_018974) [94] using the simple method of Simmons and Ochoterena [95]. Phylogenetic analyses were performed using Bayesian inference (BA) with MrBayes v3.2.2 (MrBayes, RRID:SCR_012067) [96], maximum parsimony analysis (MP) with PAUP v4.0b10 (PAUP, RRID:SCR_014931) [97], and maximum likelihood analysis with IQ-TREE v1.6.12 (IQ-TREE, RRID:SCR_017254) [98]. For BA and ML, sequence and gap data were treated as separate partitions, applying the GTR2 model on the binary partition. Prior to BA, the model of molecular evolution was determined with Modeltest v.3.5 [99] under the Akaike information criterion. The basic model parameters—the distribution of rates among sites and the number of different substitution rates—were set as priors for the partition of DNA sequences, otherwise, the default settings were used. Chains were computed for 1–2.5 million generations, sampling every 1000th tree, until all parameters indicated that convergence was reached. The first 25% of the trees were discarded as burn-in, and the rest of the trees was summarized. Models and the number of generations needed for particular datasets are summarized in Table S5. For MP analyses, heuristic searches were performed with 100 random addition sequences and TBR branch swapping, saving no more than 100 trees with length greater than or equal to 1 per replicate. Bootstrapping was done with 1000 replicates, but without branch swapping. For ML analyses, the best fitting model of molecular evolution was determined using the Akaike information criterion in the ModelFinder [100] tool of IQ-TREE (see Table S5). The ultrafast bootstrap was performed in IQ-TREE with 1000 replicates.

### 4.5. Treatment of Paralogs/Pseudogenes

Several NA samples of *Chionoracium* showed more than the two gene variants expected for heterozygous diploids with *gsh1* as well as unusually high numbers of substitutions or indels. A larger number of clones (6–22) was sequenced for each of them, and phylogenetic analyses were performed separately for *Chionoracium* to infer their diversity and positions in the tree. The clones were inspected for indications of pseudogenization (e.g., loss of exons, unusual splice sites, stop codons) in BioEdit v7.3 [93].

### 4.6. Molecular Dating

A dated molecular phylogeny of the tribe Cichorieae based on *ITS* sequences was published by Tremetsberger et al. [61]; this dataset was expanded with additional *ITS* and *ETS* sequences. The taxon sampling of the somewhat overrepresented subtribe Hypochaeridinae was reduced to 6 (out of 17) species to keep a single species per genus. *ETS* sequences were added because of the higher resolution of species relationships in subgenera *Hieracium* and *Chionoracium*. *ETS* sequences were concatenated to the corresponding *ITS* sequences and treated as missing data for other Cichorieae. No indel coding was performed for this dataset in order not to overestimate divergences among distantly related groups.

We used the fossil pollen based prior densities constructed by Tremetsberger et al. [61] and refined by Kilian et al. [101] to calibrate the ages of three divergence events within the Cichorieae. The *Cichorium intybus* type pollen (early Miocene, 22–28.4 mya) was used to calibrate the stem node of Cichorieae (*Cichorium*-type node). Following Kilian et al. [101], the calibration density was implemented as an exponential distribution with a hard lower bound of 22 mya. To capture the age range of the fossil deposit, the mean was set to 9.24 mya, which places the median of the probability at the upper age of the fossil’s deposit at 28.4 mya and the upper limit of the 95% confidence interval at 49.68 mya. The pollen of *Sonchus oleraceus*-type (late Miocene: min. 5.4 mya) is found exclusively in Hyoseridinae and must thus be assigned to the basal branch of this clade. Consequently, we specified a temporal constraint on the divergence of Hyoseridinae and its sister subtribe Crepidinae (*Sonchus*-type node) with a lower bound of 5.4 mya and an upper bound of 22 mya and used these two bounds to parametrize a uniform distribution. This upper bound is older than the 17.5 mya that corresponds to the uppermost estimate of 95% HPD for the focal node in Tremetsberger et al. [61]. The *Scorzonera hispanica*-type is found exclusively within certain species of *Scorzonera*, including *S. hispanica* and *S. suberosa*, therefore the fossil corresponding to this pollen type (middle Pliocene: min. 3.4 mya) was assigned in our tree to the basal branch of that clade. This translates into a constraint on the divergence of the *S. hispanica* and *S. suberosa* clade with its sister species *Tragopogon porrifolius* (*Scorzonera*-type node), with the fossil’s age as a lower bound and an upper bound of 22 mya and modeled with a uniform distribution. Again, the upper bound is slightly older than the 17.0 mya from the 95% HPD in Tremetsberger et al. [61]. At its core, Bayesian inference strengthens our trust in a hypothesis in the light of new pieces of evidence. This idea allowed us to restrain the temporal setting associated with the uniform distributions by leveraging results from the literature. Although it had the effect of concentrating the probability density, we still obtained wide ranges for the priors, which were nevertheless more informative than the lower bound of 100 mya used in Tremetsberger et al. [61]. We propagated the minimum bound of the *Cichorium*-type node onto the maximum bound of the *Sonchus*-type and *Scorzonera*-type nodes. This procedure enforces the constraint that ancestral nodes are older than descendent nodes and makes some topologies less probable [102].

The time calibrated dataset was analyzed in a Bayesian framework within the BEAST 2 v2.6.3 (BEAST2, RRID:SCR_017307) software platform [103,104]. We carried out a Bayesian model fitting for selecting the best combination of the features that make up the likelihood model, namely the best combination of branch rates, site and tree models. We assessed three branch-rates models that differ in the number of distinct rates that are allowed in the tree and in the underlying distribution of rates on each branch, namely the constant-rate model (STRICT clock), the uncorrelated exponential clock (UCED) and the uncorrelated lognormal clock (UCLN). Because Bayes factor comparisons (see below) favored models that sampled from a lognormal distribution over other branch-rates models, we reanalyzed all combinations that included the UCLN clock with the optimized relaxed clock (ORC) [105] parametrized with a lognormal distribution (hereafter ORC-LN clock), from the BEAST 2 plug-in ORC v1.0.2.

For all branch-rates models, we used the default setting on all priors except for the clock rate priors for which we specified a range constraint of [0.001–0.1] and a starting value of 0.01 substitutions per site per million years. As for the tree branching model, we evaluated the birth-death and the Yule processes with default hyperpriors (i.e., uniform). Tree branching model parameters were estimated during the analyses. Finally, we considered two site models to compute the sequence likelihood, namely the GTR + Γ4 + I nucleotide substitution model which was the best fitting model as determined using the AICc estimator in the ModelFinder tool and substitution model averaging provided by the bModelTest v1.2.1 plug-in for BEAST 2 [106], which implements reversible-jump Monte Carlo Markov Chain (MCMC) between time-reversible site models for nucleotides. All parameters pertaining to the general time reversible (GTR) site model were estimated during the MCMC search save the GT rate. The starting values for the shape of the gamma distribution, the proportion of invariant sites and rates were set to the estimates from ModelFinder.

Overall, we obtained sixteen combinations of features of the likelihood model. For each combination, we ran a single chain for 50 × 10^6^ generations, sampling every 5000 generations and discarded 10% of the trees as burn-in. Maximum clade credibility (MCC) trees were summarized with TreeAnnotator v.2.4.5 [103]. Tracer v.1.7.2 (Tracer, RRID:SCR_019121) [107] was used to determine the degree of mixing, shape of probability density distributions, median and HPD intervals for the relevant parameters. Adequacy of sampling was assessed via effective sampling sizes (ESS), which always exceeded 200 for the investigated statistics, and apparent mixing. Once we had ascertained that all runs were convergent and sampling was sufficient, we used the same settings to compare the feature combinations by computing their marginal likelihoods with nested sampling (NS) [108] within the NS v1.1.0 plug-in for BEAST2. We selected the most appropriate combination using Bayes factor (BF) comparisons. We obtained a standard deviation below 2.7, sufficient to discriminate the best combination, using 90 particles, a chain length of 50 × 10^6^ generations, a subchain length of 10,000 and an epsilon value of 10^−6^. We considered ln(BF) values above 5 to indicate that one model was significantly favored over another [109]. These analyses were rerun without any data (i.e., sampling from the marginal prior) to estimate the distributions of the effective priors.

For the Bayes factor favored combination, we obtained the final chronogram by running three additional independent MCMC chains for the same length as previously and combined their log outputs with the LogCombiner tool in the BEAST 2 platform, applying the same burn-in setting as before. Consistency between runs was used as a major check on MCMC convergence.

## 5. Conclusions

Combined evidence from seven molecular markers shows that native American hawkweeds are derived from *Hieracium* subgen. *Hieracium*, which is paraphyletic with respect to *H.* subgen. *Chionoracium*. *Chionoracium* originated from eastern European hawkweeds, increased in genome size, migrated via Beringia, and colonized the New World from north to south. South American species, which are investigated here for the first time with molecular markers, underwent the most recent diversification. An exception from the gradual speciation and colonization are closely related species from Alaska and Patagonia, which can be best explained by long distance dispersal.

## Figures and Tables

**Table 1:** Species relationships of subgen. *Chionoracium* revealed by different markers ^1^.

Species Clades	Lineage	ptDNA	*ITS*	*ETS*	*ITS*+ *ETS*	*5S-NTS*	*gsh1*	*sqs*	Comb.
*H. longiberbe*, *H. scouleri*	1	+++	+	–	+++	–	+	+	+++
*H. longipilum*, *H. scabrum*, *H. venosum*, *H. horridum*, *H. argutum*, *H. albiflorum*, *H. bolanderi*	2	++	–	–	–	–	–	–	–
*H. longipilum*, *H. scabrum*, *H. venosum* (+ *H. gronovii*, *ETS* and *5S-NTS* only)	2a/2a*	–	+++	++	+++	+++	+	–	+++
*H. horridum*, *H. argutum*, *H. albiflorum*, *H. bolanderi*	2b	+++	+++	–	+++	–	–	–	+++
*H. albiflorum*, *H. bolanderi*	2c	–	+++	–	+++	+++	+	+++	+++
*H. mexicanum*, *H.* cf. *guatemalense*, *H. irasuense*, *H. trichodontum*, *H. stachyoideum*, *H. glaucifolium*, *H. patagonicum*, *H.* aff. *asplundii*, *H. frigidum*	3/3*	+++	++	+++	+++	–	++	+	+++
*H. mexicanum*, *H. irasuense*	3a	+++	–	–	–	–	–	–	–
*H. trichodontum*, *H.* aff. *asplundii*	3b/3b*	+++	–	–	–	–	–	–	++
*H. glaucifolium*, *H. patagonicum*	3c	–	+++	++	+++	++	n.d.	+++	+++
*H. carneum*, *H. fendleri*	4	–	+++	+++	+++	–	–	–	+++
*H. antarcticum*, *H. gracile* (+ *H. triste*, *ETS* and *5S-NTS* only)	5	–	+++	+	++	+++	++	+	+++
*H. canadense*, *H. umbellatum* (+ *H. eriophorum*)	x	+++	+++	+++	+++	n.d.	+++	–	n.d.

^1^ Monophyletic species groups are listed; lineage numbers are included in the tree figures and explained in the text. The number of ‘+’ corresponds to the number of analyses (Bayesian Analysis [BA], MP, ML) supporting the respective branches with posterior probabilities >0.94 and bootstrap support >70. n.d.: not determined. –: not significant. The asterisk (*) indicates groups of slightly different species compositions.

**Table 2:** *Gsh1* gene duplications and pseudogenization in four accessions of *Chionoracium* ^1^.

Accession, Clone	Sequence Features				
	AG	TG	Stop	Poly-T	Exon Missing
*H. horridum* c1–5, 7, 12–15, 17–18, 20	+				
*H. horridum* c6, 8, 9, 11	+		+		
*H. horridum* c16, 19	+	+	+		
*H. argutum* c1, 10, 16, 22	+				
*H. argutum* c2, 3, 6, 8–9, 13–18, 23	+	+			
*H. argutum* c4, 7, 20–21		+			
*H. argutum* c5, 11–12				+	
*H. argutum* c19		+		+	
*H. albiflorum* H908 c1	(+)				
*H. albiflorum* H908 c2, 4–5	+				
*H. albiflorum* H908 c3, 6					
*H. scouleri* var. *albertinum* c1–2, 4–10					
*H. scouleri* var. *albertinum* c3	+				+
*H. scouleri* var. *albertinum* c11					+

^1^ Unusual sequence features that may be indicative of pseudogenization are summarized. AG: Additional AG adjacent to AG splice site of exon 13. TG: Corrupted splice site (TG) of exon 12. Stop: Stop codon in exon 13. Poly-T: Unusually long poly-T (31–35 Ts) in intron 13. Exon missing: Exon 12 and parts of adjacent introns 11 and 12 missing. *Hieracium albiflorum* H908 clone 1 is a partial sequence nearly identical to clones 2, 4, and 5.

**Table 3:** Dated divergence events ^1^.

Node Number	Description	Age Estimates Mya (Range)
1	Hieraciinae	7.45 (3.95–12.07)
2	Hieraciinae without *H. intybaceum*	4.99 (2.74–7.94)
3	*Hieracium* subgenera *Hieracium* and *Chionoracium*	3.14 (1.78–5.01)
4	*H.* subgen. *Hieracium*, W European clade	1.13 (0.46–2.11)
5	*H.* subgen. *Hieracium*, E European clade & *Chionoracium*	2.24 (1.29–3.47)
6	Divergence in the *H. umbellatum* clade (incl. *H. canadense*)	0.22 (0.04–0.52)
7	wNA species, lineages 1, 2b & 2c	1.58 (1.19–3.16)
8	*H. longiberbe*/*H. scouleri* (wNA, lineage 1)	0.82 (0.20–1.61)
9	*H. horridum*/*H. argutum*/*H. albiflorum*/*H. bolanderi* (wNA, lineage 2b)	1.05 (0.50–1.79)
10	*H. albiflorum*/*H. bolanderi* (wNA, lineage 2c)	0.04 (0.0–0.17)
11	all other species of *H.* subgen. *Chionoracium*	1.59 (0.89–2.45)
12	*H. antarcticum*/*H. gracile* (sSA, nNA/wNA, lineage 5)	0.40 (0.08–0.90)
13	*H. longipilum*/*H. venosum*/*H. scabrum* (eNA, lineage 2a)	0.36 (0.03–0.41)
14	*H. carneum*/*H. fendleri* (wNA, lineage 4)	0.36 (0.07–0.82)
15	all SA species (except *H. antarcticum*) and *H. mexicanum* (CA)	0.79 (0.40–1.31)
16	*H. glaucifolium*/*H. patagonicum* (sSA, lineage 3c)	0.20 (0.04–0.45)

^1^ The age estimates were inferred using the Bayes factor favored modelling scheme, combining the optimized relaxed clock parametrized with a lognormal distribution (ORC-LN) model with the birth-death process and the GTR + Γ4 + I model (Table S3). The node numbers refer to Figure 7. mya: million years ago.

## Data Availability

Sequence data have been submitted to the GenBank database (accession numbers MZ322103–MZ322303, MZ329404–MZ32942).

## Supplementary Materials

The following supporting information can be downloaded at: https://www.mdpi.com/article/10.3390/plants11192584/s1, Figure S1: Phylogenetic analyses of subgen. *Chionoracium* based on nrDNA (*ITS*); Figure S2: Phylogenetic analyses of subgen. *Chionoracium* based on nrDNA (*ETS*); Figure S3: Gene tree of subgen. *Chionoracium* including paralogs/pseudogenes of *gsh1*; Table S1: Genome size measurements of native American hawkweeds; Table S2: Classification and distribution of *Hieracium* subgen. *Chionoracium* species; Table S3: Log marginal likelihoods and median age estimates for the Hieraciinae node; Table S4: Details of sample origins; Table S5: Overview of markers, datasets, and phylogenetic analyses.

## Appendix A. Vouchers and GenBank Accession Numbers

For details of sample origins, see Table S4.

***Species*** + author, **accession**, *origin and herbarium voucher*, GenBank numbers for *trnT-*

*trnL*, *trnV-ndhC*, *ITS*, *ETS*, *sqs*, *gsh1*, *5S-NTS*.

**Ingroup (*Hieracium* subgen. *Chionoracium*): *Hieracium albiflorum*** Hook, **H908**, *USA: Idaho (PRA)*, MZ322157, MZ322172, MZ329404, MZ322103, MZ322196, MZ322223–MZ322228, MZ32213; **albifl**, *B&T World Seed*, AY573352, MZ322173, MZ329405, MZ322104, MZ322197, MZ322229, –; ***H. antarcticum*** D’Urv., **ant.2**, *Chile: Patagonia (GLM 155579)*, AY573350, MZ322174, MZ329406, MZ322105, MZ322198, MZ322230, MZ322133; ***H. argutum*** Nutt., **H905**, *USA: California (PRA)*, MZ322158, MZ322175, MZ329407, MZ322106, MZ322199, MZ322231–MZ322253, MZ322134; ***H.* aff. *asplundii*** Sleumer, **asp.Bol.2**, *Bolivia: Prov. Murillo (M)*, AY573348, MZ322176, MZ329408, MZ322107, MZ322200, MZ322254, MZ322135; ***H. bolanderi*** Gray, **4475**, *USA: California (ID)*, MZ322159, MZ322177, MZ329409, MZ322108, MZ322201, MZ322255, MZ322136; ***H. carneum*** Greene, **H.carneum.1**, *USA: Arizona (PRA)*, AY573351, MZ322178, MZ329410, MZ322109, MZ322202, MZ322256, MZ322137; ***H. fendleri*** Sch.Bip., **FEND1**, *USA: Arizona (PRA)*, MZ322160, MZ322179, MZ329411, MZ322110, MZ322203, MZ322257, MZ322138; ***H. frigidum*** Wedd., **Chi.Ecu**, *Ecuador: Prov. Tungurahua (PRA)*, AY573346, MZ322185, MZ329417, MZ322118, MZ322210, MZ322282, MZ322145; ***H. glaucifolium*** Poepp. ex Froelich, **bg53**, *Chile (M)*, MZ322161, MZ322180, MZ329412, MZ322111, MZ322204, MZ322258, MZ322139; ***H. gracile*** Hook, **gracile**, *USA: Idaho (PRA)*, MZ322162, MZ322181, MZ329413, MZ322112, MZ322205, MZ322259, MZ322140; ***H. gronovii*** L., **4371**, *USA: Michigan (ID)*, –, –, –, MZ322113, –, –, MZ322141; ***H.* cf *guatemalense*** Standl. et Steyerm., **GUA3**, *Guatemala: Quetzaltenango (PRA)*, MZ322163, MZ322182, MZ329414, MZ322114, MZ322206–MZ322207, MZ322260–MZ322261, MZ322142; ***H. horridum*** Fr., **4781**, *USA: California (WTU)*, MZ322164, MZ322183, MZ329415, MZ322115, MZ322208, MZ322262–MZ322280, MZ322143; ***H. irasuense*** Benth., **4772**, *Mexico: Chiapas (MSC)*, –, –, –, MZ322116, –, –, –; **ira2-9**, *Guatemala: San Marcos (PRA)*, MZ322165, MZ322184, MZ329416, MZ322117, MZ322209, MZ322281, MZ322144; ***H. longiberbe*** Howell, **4783,** *USA: Oregon (ID)*, MZ322166, MZ322186, MZ329418, MZ322119, MZ322211–MZ322212, MZ322283–MZ322284, MZ322146; ***H. longipilum*** Torr. ex Hook, **4376,** *USA: Wisconsin (ID)*, MZ322167, MZ322187, MZ329419, MZ322120, MZ322213, MZ322285, MZ322147; ***H. mexicanum*** Less., **1093_TA**, *Guatemala: San Marcos (PRA)*, MZ322168, MZ322188, HQ131824, MZ322121, HQ131845, HQ131789, MZ322148; **4773**, *Mexico: Mexico (MSC)*, –, –, –, MZ322122, –, –, –; ***H.* cf *patagonicum*** Hook, **pat.Chi**, *Chile: Refugio Redeto culture no. 94-55 (M)*, AY573349, MZ322189, MZ329420, MZ322123, MZ322214, –, MZ322149; ***H. scabrum*** Michx., **H911**, *USA: Michigan (PRA)*, MZ322169, –, MZ329421, MZ322124, –, MZ322286–MZ322287, –; **1002**, *USA: Michigan (PRA)*, MZ322170, MZ322190, HQ131825, MZ322125, HQ131844, HQ131790–HQ131791, MZ322150; ***H. scouleri*** Hook **var. *albertinum*** (Farr)G.W. Douglas & G.A.Allen, **H903**, *USA: Idaho (PRA)*, MZ322171, MZ322191, MZ329422, MZ322126, MZ322215–MZ322216, MZ322288–MZ322298, MZ322151; ***H. s.* var. *scouleri***, **4788**, *USA: Idaho (ID)*, –, –, –, MZ322127, –, –, MZ322152; ***H. stachyoideum*** Arv.-Touv., **sty.Bol**, *Bolivia: Prov. Murillo (M)*, AY573347, MZ322192, MZ329423, MZ322128, MZ322217, MZ322299, MZ322153; ***H. trichodontum*** (Schulz-Bip.)Arv.-Touv., **tri.Bol**, *Bolivia: Prov. Camacho (M)*, AY573345, MZ322193, MZ329424, MZ322129, MZ322218–MZ322219, MZ322300–MZ322301, MZ322154; ***H. triste*** Willd. ex Spreng., **4792**, *USA: Alaska (WTU)*, –, –, –, MZ322130, –, –, MZ322155; ***H. venosum*** L., **venosum**, *USA: Michigan (MICH, GLM 141161)*, AY573354, MZ322194, MZ329425, MZ322131, MZ322220–MZ322221, MZ322302–MZ322303, MZ322156. **Outgroup (*Hieracium* subgen. *Hieracium*, *Hispidella*, *Pilosella*): *Hieracium alpinum*** L., **alp.Ukr**, *Ukraine: Chornohora Mts. (PRA)*, AY512556, JX129538, AJ633429, EU867634, JX129606–JX129607, MK465558–MK465559, MW328890; **alp.Boa.2**, *Romania: Munţii Rodnei (PRA)*, EU867711, JX129539, MZ329426, EU867633, JX129608–JX129609, MK465560–MK465561, –; ***H. canadense*** Michx., **canad.**, *B&T World Seed (GLM 157756)*, AY573341, JX129546, MZ329427, EU867638, JX129631–JX129632, MK465576–MK465577, –; ***H. eriophorum*** St.-Amans, **1221/1**, *France: dépt.* *Landes (PRA)*, EU867751, JX129550, MW315935, EU867639, JX129638–JX129639, MK465585, MW328891; **1222/2**, *France: dépt. Landes (PRA)*, EU867762, JX129551, MW315936, EU867641, JX129640–JX129641, MK465586, –; ***H. intybaceum*** All., **Inb.Kaer**, *Austria: Kärnten (M)*, AY573323, JX129561, KM372113, EU821370, JX129745, MK465606–MK465607, MW328892; **1069/1**, *Italy: Trentino (PRA)*, JX129600, JX129560, HQ131821, KM372006, HQ131846–HQ131847, MK465605, –; ***H. kittanae*** Vladimirov, **1228/2**, *Bulgaria: Central Rhodope Mts. (PRA)*, EU867741, JX129562, MW315937, EU867622, JX129661–JX129662, MK465608–MK465609, MW328893; ***H. laniferum*** Cav., **lanif2**, *Spain: prov. Tarragona (PRA)*, MK523486, MZ322195, MW315938, MK523499, MK523519, MK523537, MW328894; ***H. lucidum*** Guss., **H. lucidum**, *Italy: Sicily (PRA)*, EU867760, JX129566, MW315939, EU867593, JX129672–JX129673, MK465618, MW328895; ***H. petrovae*** Vladimirov & Szeląg, **1229**, *Bulgaria: Central Rhodope Mts. (PRA)*, EU867740, JX129572, MW325265, EU867625, MK523523–MK523524, MK465631–MK465632, MW328949; ***H. porrifolium*** L., **1052/9**, *Austria: Kärnten (PRA)*, EU867730, JX129578, HQ131823, EU867631, HQ131843/JX129701, HQ131785–HQ131786, MW328896; ***H. prenanthoides*** Vill., **1252**, *France: dépt. Hautes Alpes (PRA)*, EU867745, JX129579, MW315942, EU867579, JX129702–JX129703, MK465644, MW328897; ***H. recoderi*** De Retz, **1174/4**, *Spain: Catalunya (PRA)*, EU867721, JX129584, KM372114, EU867603, MK523525–MK523526, MK465656, MW328898; ***H. sparsum*** Friv., **1251/1**, *Bulgaria: Sofia (PRA)*, EU867710, JX129587, MW315944, EU867626, JX129720–JX129721, MK465660–MK465661, –; **spa.sst.2**, *Bulgaria: Pirin Mts. (PRA)*, AY573333, JX129588, AJ633431, EU867628, JX129722–JX129723, MK465662–MK465663, MW328899; ***H. stelligerum*** Froel., **1233/1**, *France: dépt. Ardèche (PRA)*, EU867755, JX129589, MW315945, EU867597, JX129724–JX129725, MK465664, MW328900; ***H. tomentosum*** L., **1066/8**, *France: dépt. Alpes Maritimes (PRA)*, EU867731, JX129590, KM372115, EU867596, JX129726–JX129727, MK465665, MW328901; ***H. transylvanicum*** Heuff., **tra.Boa**, *Romania: Munţii Rodnei (PRC)*, AY512557, JX129591, MW315947, EU867571, JX129728–JX129729, MK465666–MK465667, MW328902; **1077/7**, *Ukraine: Oblast Zakarpatska (PRA)*, EU867743, JX129592, MW587348, EU867572, JX129730–JX129731, MK465668, MW328962; ***H. umbellatum*** L., **1021/1**, *Poland: Województwo pomorskie (PRA),* EU867752, JX129593, HQ131822, EU867642, HQ131841–HQ131842, HQ131787–HQ131788, MW328904; **um.AM.1**, *Germany: Upper Lusatia (GLM 46889)*, AY573335, JX129594, KM372116, EU867644, JX129732, MK465669–MK465670, MW328905; ***H. vranceae*** Mráz, **vran.1-1**, *Romania: Vrancea (PRC),* MK523491, –, MW315951, MK523515, MK523529–MK523530, MK523547–MK523548, MW328907; ***Hispidella hispanica*** Barnades ex Lam., **His.his.2**, *Spain: Sierra de Guadarrama (PR no CN 2460)*, AY573355, JX129534, KM372107, EU821365, JX129601–JX129602, HQ131797/JF519822, MW328924; ***P. argyrocoma*** (Fr.)F.W.Schultz & Sch.Bip., **agy.Gra**, *Spain: prov. Granada (culture H11, M)*, AY573320, JX129536, KM372108, KM372001, JX129605, –, MW328914; ***Pilosella lactucella*** (Wallr.)P.D.Sell & C.West; **lac.Jon.1**, *Germany: Jonsdorf (GLM 140619)*, AY192669, JX129535, KM372109, KM372002, JX129603, HQ131793/HQ131795, MW328918; ***P. pseudopilosella*** (Ten.)Soják, **TU308/2**, *Spain: Sierra Nevada (PRA)*, JX129599, JX129537, KM372110, KM372003, MZ322222, –, –.
