# Supplementary material for: A Multigene Phylogeny of Native American Hawkweeds (Hieracium Subgen. Chionoracium, Cichorieae, Asteraceae): Origin, Speciation Patterns, and Migration Routes
Source: Plants (Basel). 2022 Sep 30;11(19):2584. doi: 10.3390/plants11192584 (PMC9571344; doi:10.3390/plants11192584)
Supplement: Supplementary file 1 [file plants-11-02584-s001.zip › Figure S1_ITS_Plants.pdf]

Figure S1. **Phylogenetic analyses of subgen. *Chionoracium* based on nrDNA (*ITS*)**

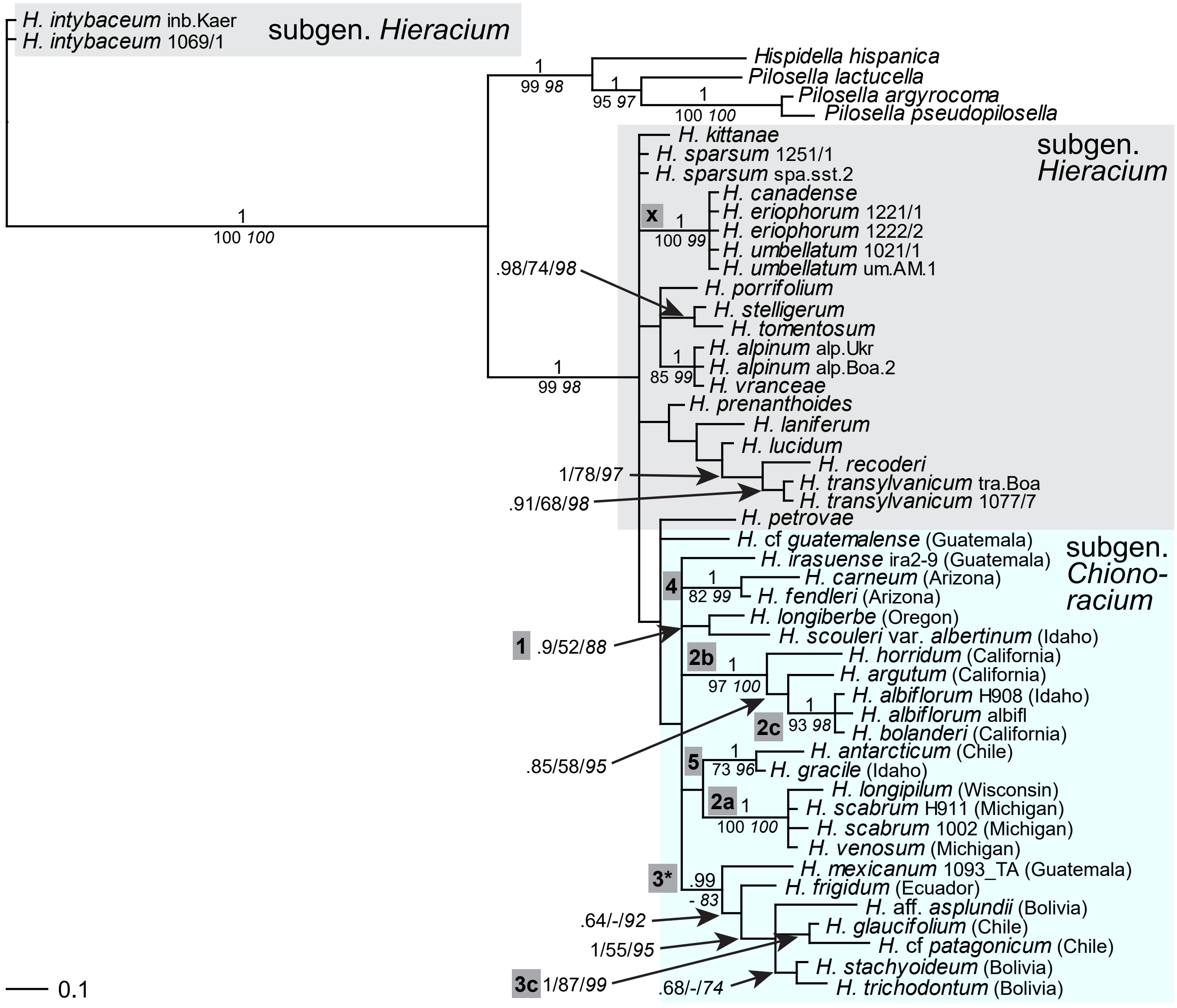

The Bayesian consensus tree is shown with posterior probabilities (pp) above branches and bootstrap support (BS) from MP (regular font) and ML analyses (italics) below branches. Support values are only given for branches, if pp was >0.94 or BS was >70 in at least one type of analysis. If more than one accession per species was analyzed, their labels are indicated after the species name. Numbers in grey boxes refer to clades of closely related species (see text and Table 1).
