# Supplementary material for: A Multigene Phylogeny of Native American Hawkweeds (Hieracium Subgen. Chionoracium, Cichorieae, Asteraceae): Origin, Speciation Patterns, and Migration Routes
Source: Plants (Basel). 2022 Sep 30;11(19):2584. doi: 10.3390/plants11192584 (PMC9571344; doi:10.3390/plants11192584)
Supplement: Supplementary file 1 [file plants-11-02584-s001.zip › Figure S3_gsh-paralogs_Plants.pdf]

**Figure S3. Gene tree of subgen. *Chionoracium* including paralogs / pseudogenes of *gsh1***

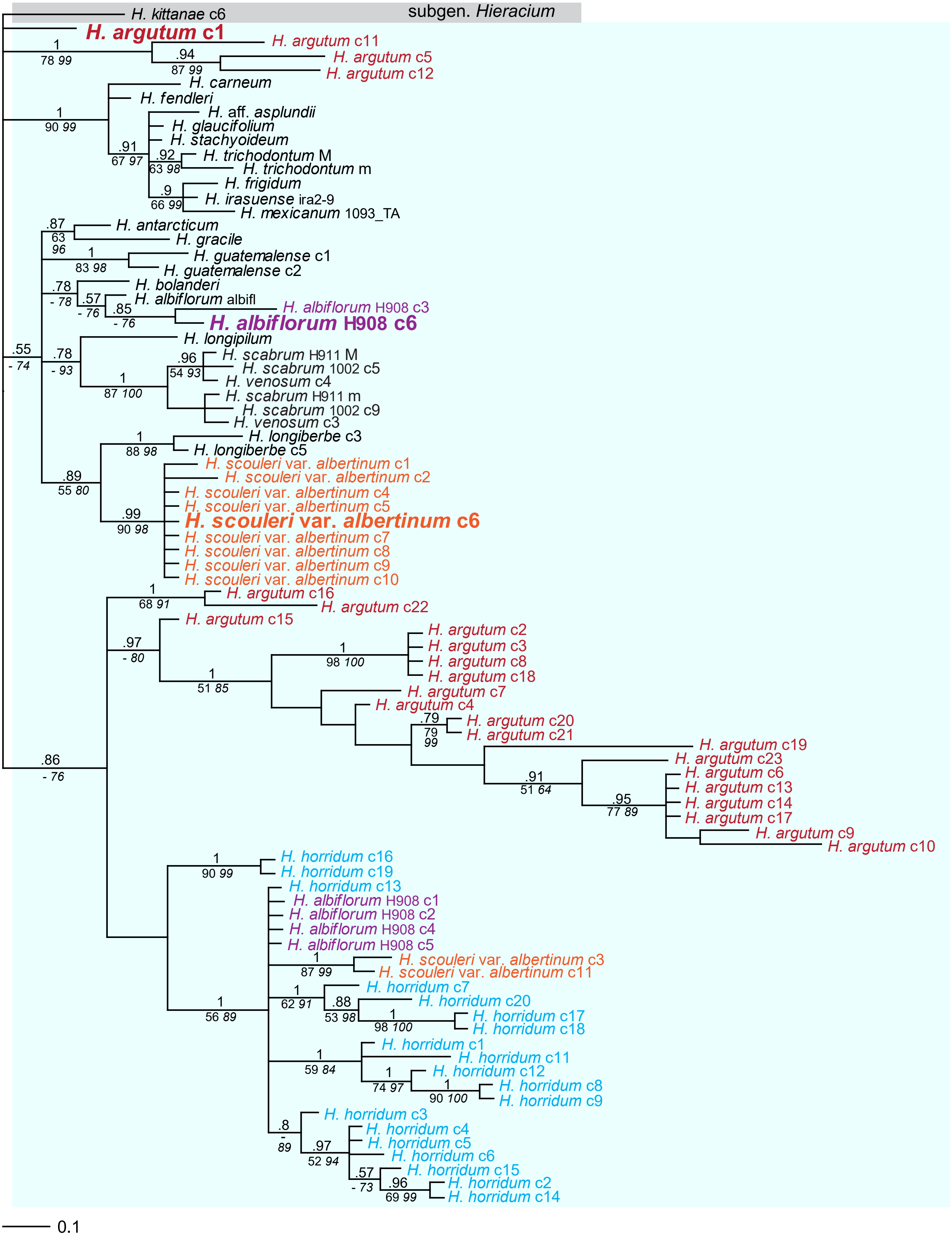


The Bayesian consensus tree is shown with posterior probabilities (pp) above branches and bootstrap support (BS) from MP (regular font) and ML analyses (italics) below branches. Support values are only given for branches, if pp was >0.94 or BS was >70 in at least one type of analysis. If more than one accession per species was analyzed, their labels are indicated after the species name. *Hieracium kittanae* clone 6 is included as outgroup (see Figure 4). Four accessions (in different colors) show a proliferation of paralogs and/or pseudogenes represented by 6–23 clones per sample. Three clones in bold are supposed to represent native copies and were included in phylogenetic analyses (Figure 4). For more explanations, see text.
