# Supplementary material for: A Multigene Phylogeny of Native American Hawkweeds (Hieracium Subgen. Chionoracium, Cichorieae, Asteraceae): Origin, Speciation Patterns, and Migration Routes
Source: Plants (Basel). 2022 Sep 30;11(19):2584. doi: 10.3390/plants11192584 (PMC9571344; doi:10.3390/plants11192584)
Supplement: Supplementary file 1 [file plants-11-02584-s001.zip › Table S1_genome_sizes_Plants.pdf]

TABLE S1. Genome size measurements of native American hawkweeds

Species	Accession	*2n*	Average 2C [pg]	2C range [pg]
H. albiflorum	H908_1		7.517	7.515–7.521
	H908_2		7.542	7.493–7.591
	H908_3		7.558	7.537–7.580
	H908 (all)		***7.539***	***7.493–7.591***
H. canadense	***canad***		***12.332***	–
H. carneum	H.carneum.1		9.515	9.416–9.606
	H.carneum.2		9.533	9.432–9.584
	H.carneum.3		9.531	9.454–9.595
	H. carneum (all)		***9.526***	***9.416–9.606***
H. guatemalense	GUA/2 + Z	18	9.227	9.193–9.253
	GUA/3 + Z		9.209	9.188–9.231
	GUA/4 + Z		9.217	9.160–9.269
	GUA/5 + Z	18	9.262	9.193–9.318
	GUA/6 + Z		9.248	9.236–9.264
	GUA (all)		***9.233***	***9.160–9.318***
H. mexicanum	***H903 (8 plants)***		***10.14***	***10.09–10.23***
H. scabrum	1002/2 + Z	18	9.922	9.861–9.959
	1002/4 + Z		9.942	9.926–9.959
	1002 (all)		***9.932***	***9.861–9.959***

Accession: several plants from the same population per species, individual measurements and average for the population (3 measurements, if not indicated otherwise); 2*n* – chromosome counts (2*n* = 2x = 18). The accession of *H. canadense* is triploid according to its genome size (one measurement only).
