# Supplementary material for: A Multigene Phylogeny of Native American Hawkweeds (Hieracium Subgen. Chionoracium, Cichorieae, Asteraceae): Origin, Speciation Patterns, and Migration Routes
Source: Plants (Basel). 2022 Sep 30;11(19):2584. doi: 10.3390/plants11192584 (PMC9571344; doi:10.3390/plants11192584)
Supplement: Supplementary file 1 [file plants-11-02584-s001.zip › Table S2_classification_distribution_Plants.pdf]

TABLE S2. Classification and distribution of *Hieracium* subgen. *Chionoracium* species. *Hieracium umbellatum* and *H. canadense* of subgen. *Hieracium*, which occur (also) in North America are included as well. Sectional classification is shown after the species name; species assigned to the same section are in matching colors.

Species	Distribution
H. albiflorum (Stenotheca)	western North America with a few outposts in central and eastern North America
H. antarcticum (Aurelliformia)	southern Chile and Argentina
H. argutum (Stenotheca)	California
H. aff. *asplundii*	Bolivia
H. bolanderi (Cynoglossoidea)	Oregon, California and Mexico (Baja California)
H. canadense	Canada, northern U.S.A.
H. carneum (Crepidispermum)	Arizona, New Mexico, Texas, northern Mexico
H. fendleri (Crepidispermum)	southwestern U.S.A., Mexico, rare in Guatemala
H. frigidum (Aurelliformia)	Colombia, Ecuador, Venezuela
H. glaucifolium (Piloselliformia)	southern Chile and Argentina
H. gracile (Aurelliformia)	western Canada, western U.S.A.
H. gronovii (Stenotheca)	eastern and central North America, Carribean
H. cf. *guatemalense*	Guatemala
H. horridum (Cynoglossoidea)	California, Oregon, Nevada
H. irasuense (Thyrsoidea)	central America
H. longiberbe (Cynoglossoidea)	Oregon, Washington
H. longipilum (Cynoglossoidea)	central and eastern U.S.A., Quebec
H. mexicanum (Thyrsoidea)	southern Mexico, Guatemala
H. patagonicum (Piloselliformia)	southern Chile and Argentina, Falkland Islands
H. scabrum (Stenotheca)	southeastern Canada, eastern U.S.A.
H. scouleri var. *albertinum (Cynoglossoidea)*	western Canada, western U.S.A.
H. scouleri var. *scouleri (Cynoglossoidea)*	western Canada, western U.S.A.
H. stachyoideum (Mandonia)	Bolivia, northern Argentina
H. trichodontum (Aurelliformia)	Bolivia
H. triste (Aurelliformia)	Aleutian Islands, Alaska, western Canada, Russian Far East
H. umbellatum	Europe, Asia, North America
H. venosum (Stenotheca)	eastern North America with a few outposts in central North America and Alaska
