# Supplementary material for: A Multigene Phylogeny of Native American Hawkweeds (Hieracium Subgen. Chionoracium, Cichorieae, Asteraceae): Origin, Speciation Patterns, and Migration Routes
Source: Plants (Basel). 2022 Sep 30;11(19):2584. doi: 10.3390/plants11192584 (PMC9571344; doi:10.3390/plants11192584)
Supplement: Supplementary file 1 [file plants-11-02584-s001.zip › Table S3_dating_models_Plants.pdf]

TABLE S3. Log marginal likelihoods and median age estimates for the Hieraciinae node

Combination number	Ln(BF)	Log marginal likelihood	SD	H	Branch rate model	Tree branching model	Site model	Hieraciinae age estimate (mya)
1	-	-13802	2.6	599	ORC-LN	B&D	GTR+Γ+I	7.5 [3.9–12.3]
2	5	-13807	2.6	607	UCLN	B&D	GTR+Γ+I	7.6 [4.21–12.1]
3	6	-13808	2.6	617	UCED	B&D	GTR+Γ+I	8.5 [3.4–15.3]
4	7	-13809	2.7	609	UCLN	Y	GTR+Γ+I	8.3 [4.5–13.2]
5	9	-13811	2.6	611	ORC-LN	Y	GTR+Γ+I	8.1 [4.4–13.0]
6	18	-13820	2.6	630	UCED	B&D	average	8.8 [3.4–15.6]
7	19	-13821	2.1	630	UCED	Y	GTR+Γ+I	10.2 [4.9–16.7]
8	21	-13823	2.6	599	ORC-LN	B&D	average	7.6 [4.1–11.8]
9	25	-13827	2.6	605	UCLN	B&D	average	7.6 [4.3–12.4]
10	29	-13831	2.6	608	UCLN	Y	average	8.2 [4.6–13.3]
11	29	-13831	2.6	609	ORC-LN	Y	average	8.1 [4.4–13.1]
12	30	-13832	2.7	642	UCED	Y	average	10.5 [5.4–16.9]
13	39	-13841	2.6	551	STRICT	B&D	GTR+Γ+I	5.6 [4.1–7.5]
14	47	-13849	2.4	557	STRICT	Y	GTR+Γ+I	5.7 [4.3–7.8]
15	55	-13857	2.6	567	STRICT	Y	average	5.8 [4.3–7.8]
16	56	-13858	2.6	551	STRICT	B&D	average	5.6 [4.1–7.4]

The computations were performed using nested sampling implementation from the NS v1.1.0 plug-in for BEAST2, with a MCMC chain length of $50x10^6$ generations, a subchain length of 10000, an epsilon value of 10^{-6} and 90 particles. We report estimates for the likelihood and its standard deviation (SD). The value of the information parameter (H) was used to assess the number of particles required for achieving sufficient discriminatory power to obtain reliable evidence in favour of one of the models combination. ESS were >1000 in every analysis. Competing combinations are ordered by decreasing likelihood. The Bayes factor (BF) is computed as the difference in marginal likelihood with the favoured combination. The combinations differ according to the components of the likelihood model, namely the branch rate model (UCLN, UCED, STRICT, ORC-LN clocks), the branching model (Yule [Y], birth-death [B&D] processes) and the site model (GTR + Γ4 + I , model averaging [average] nucleotide substitution models). We interpret a ln(BF) value above 5 to provide an overwhelming support for one combination over the competing alternative. Estimates for the Hieraciinae divergence are the median ages and 95% Highest Posterior Density (HPD).
