# Supplementary material for: A Multigene Phylogeny of Native American Hawkweeds (Hieracium Subgen. Chionoracium, Cichorieae, Asteraceae): Origin, Speciation Patterns, and Migration Routes
Source: Plants (Basel). 2022 Sep 30;11(19):2584. doi: 10.3390/plants11192584 (PMC9571344; doi:10.3390/plants11192584)
Supplement: Supplementary file 1 [file plants-11-02584-s001.zip › Table S4_sample_origins_Plants.pdf]

Table S4. Details of sample origins.

Species	Identifier	Origin and voucher
Hieracium* subgen. *Chionoracium		
Hieracium albiflorum	H908	USA: Idaho, Bouner County, Talache, leg. L.M. Wilson July 2001 (PRA – plants cultivated from seeds)
	albifl	B&T World Seed, http://www.b-and-t-world-seeds.com/index.html
H. antarcticum	ant.2	Chile: Patagonia National Park, Torres del Paine, leg. F. Müller 2000 (GLM 155579)
H. argutum	H905	USA: California, Monterey County, Big Sur, leg. L.M. Wilson 20 June 2002 (PRA – plants cultivated from seeds)
H. aff. *asplundii*	asp.Bol.2	Bolivia: Prov. Murillo, Dpto La Paz, Beck 14779 (M)
H. bolanderi	4475	USA: California, Del Norte County, leg. Roche & Korfhage R-2024 (ID)
H. carneum	H.carneum.1	USA: Arizona, Pima Co., Mt. Lemon, leg. L.M. Wilson June 2004 (PRA – plants cultivated from seeds)
H. fendleri	FEND1	USA: Arizona, Tucson, Rincon Mts, leg. L.M. Wilson June 2004 (PRA – plants cultivated from seeds)
H. frigidum	Chi.Ecu	Ecuador: Prov. Tungurahua, P.N. Llangantes, leg. Z. Palice 2003, det. F. Schuhwerk (PRA)
H. glaucifolium	bg53	Chile: leg. J. Grau (M)
H. gracile	gracile	USA: Idaho, Shoshone County, Freezout Mountain, leg. L.M. Wilson 22 August 2006 (PRA – plants cultivated from seeds)
H. gronovii	4371	USA: Michigan, Kalamazoo County, leg. L.M. Wilson s.n. (ID)
H. cf *guatemalense*	GUA3	Guatemala: Quetzaltenango, Santa Maria volcano, leg. K. Krak August 2005 (PRA)
H. horridum	4781	USA: California, Mono County, leg. unknown (WTU)
H. irasuense	4772	Mexico: Chiapas, leg. Villasenor et al. s.n. (MSC)
	ira2-9	Guatemala: San Marcos, Tajumulco volcano, near the summit, leg. K. Krak August 2005, det. F. Schuhwerk (PRA)
H. longiberbe	4783	USA: Oregon, Multnomah County, Oeneta Gorge, leg. L.M. Wilson 8 July 2004 (ID)
H. longipilum	4376	USA: Wisconsin, Adams County, leg. Cochrane 14234 (ID)
H. mexicanum	1093_TA	Guatemala: San Marcos, Tajumulco volcano, leg. K. Krak August 2005, det. F. Schuhwerk (PRA)
	4773	Mexico: Mexico, leg. Soule 1393 (MSC)
H. patagonicum	pat.Chi	Chile: Refugio Redeto, cultivated at Botanical Garden Munich, culture no. 94-55, voucher from 25.10.1995 (M)
H. scabrum	H911	USA: Michigan I, leg. L.M. Wilson, s.d. (PRA – plants cultivated from seeds)
	1002	USA: Michigan II, leg. L.M. Wilson, s.d. (PRA – plants cultivated from seeds)
H. scouleri var. *albertinum*	H903	USA: Idaho, Benewah County, Lindstrom, leg. L.M. Wilson 6 August 2002 (PRA – plants cultivated from seeds)
H. scouleri var. *scouleri*	4788	USA: Idaho, Idaho County, leg. unknown (ID)
H. stachyoideum	sty.Bol	Bolivia: Prov. Murillo, Dpto Cochabamba, leg. Beck 14565 & Seidel (M)
H. trichodontum	tri.Bol	Bolivia: Prov. Camacho, Dpto La Paz, leg. Beck 4233 (M)
H. triste	4792	USA: Alaska, Skagway, leg. unknown (WTU)
H. venosum	venosum	USA: Michigan, leg. Reznicek 11266 (MICH, GLM 141161)
Hieracium* subgen. *Hieracium		
Hieracium alpinum	alp.Ukr	Ukraine: Chornohora Mts. (Carpathians), Polonina Breskulska ridge, 23 July 2003, leg. P. Mráz & J. Chrtek, 0038/2003 (PRA)
	alp.Boa.2	Romania: Munţii Rodnei, glacial circle on the NE slopes of Mt. Pietrosul Mare, 5 July 2001, leg. P. Mráz, 0187/2001 (PRA)
H. canadense	canad	B&T World Seed, http://www.b-and-t-world-seeds.com/index.html (GLM 157756)
H. eriophorum	1221/1	France: dépt. Landes, Labenne: plage de Labenne Océan Sud, 27 September 2006, leg. E. Forey, 1221/2006 (PRA)
	1222/2	France: dépt. Landes, Seignosse-le-Penon, plage de Estagnols Seignosse, 27 September 2006, leg. E. Forey, 1222/2006 (PRA)
H. intybaceum	inb.Kaer	Austria: Kärnten, Turracher Höhe, S. Jagalski 4 (M)
	1069/1	Italy: Trentino, Alto Adige, Passo del Tonale, 31 August 2005, leg. J. Chrtek & P. Mráz, 1069/2005 (PRA)

H. kittanae	1228/2	Bulgaria: Central Rhodope Mts., Trigrad gorge, September 2005, leg. P. Ignatova, 1228/2005 (PRA)
H. laniferum	lanif2	Spain: prov. Tarragona, la Sénia, 4 June 2011, leg. J. Chrtek (PRA)
H. lucidum	H. lucidum	Italy: Sicily, distr. Palermo, Sferracavallo, 11 April 2007, leg. J. Chrtek et al., 0054/2007 (PRA)
H. petrovae	1229	Bulgaria: Central Rhodope Mts., Trigrad gorge, 15 October 2005, leg. V. Vladimirov, 1229/2005 (PRA)
H. porrifolium	1052/9	Austria: Kärnten, Karawanks, Bad Eisenkappel, 26 July 2005, leg. J. Chrtek & P. Mráz, 1052/2005 (PRA)
H. prenanthoides	1252	France: dépt. Hautes Alpes, La Grave, below the village, June 2003, leg. P. Mráz, 1252/2003 (PRA)
H. recoderi	1174/4	Spain: Catalunya, prov. Barcelona, Berga, 24 July 2006, leg. J. Chrtek, 1174/2006 (PRA)
H. sparsum	1251/1	Bulgaria: Sofia, Vitoša Mts., NE slope of Mt. Vitoša, 23 June 2006, leg. F. Krahulec & A. Krahulcová, 1251/2006 (PRA)
	spa.sst.2	Bulgaria: Pirin Mts., Mt. Vihren, garden culture Z. Szeląg, 0034/2006 (PRA)
H. stelligerum	1233/1	France: dépt. Ardèche, Vallon Pont d'Arc, October 2006, leg. P. Mráz, 1233/2006 (PRA)
H. tomentosum	1066/8	France: dépt. Alpes Maritimes, Roya Valley, Tende, 28 August 2005, leg. J. Chrtek & P. Mráz, 1066/2005 (PRA)
H. transylvanicum	tra.Boa	Romania: Munţii Rodnei, path from the village of Borşa to Mt. Pietrosul Mare, July 2001, leg. P. Mráz (PRC)
	1077/7	Ukraine: Oblast Zakarpatska, Marmaroski Alps, Mt. Berlebashka, 19 September 2005, leg. J. Zahradníček, 1077/2005 (PRA)
H. umbellatum	1021/1	Poland: Województwo pomorskie, Baltic coast, Jantar, 27 June 2002, leg. Z. Szeląg, 1021/2002 (PRA)
	um.AM.1	Germany: Upper Lusatia, SE Schönau-Berzdorf, slagheap, 20 August 2003, leg. S. Bräutigam (GLM 46889)
H. vranceae	Hvran_1-1	Romania: Vrancea, Cheile Tişiţei, 28 August 2013, leg. L. Filipaş & M. Bărbos (PRC)
Hispidella hispanica	His.his.2	Spain: Sierra de Guadarrama, leg. J. Pizarro & C. Navarro (PR, no CN 2460)
Pilosella argyrocoma	agy.Gra	Spain: prov. Granada, cultivated in Botanic Garden Munich, H. Merxmüller & W. Gleisner, culture H11
P. lactucella	lac.Jon.1	Germany: Jonsdorf, Oberlausitz, parking lot, June 1999, leg. S. Bräutigam (GLM 140619)
P. pseudopilosella	TU308/2	Spain: Sierra Nevada, between Granada and Pradollano, 2 June 2008, leg. T. Urfus, T. Urfus 308/2 (PRA)
