# Supplementary material for: A Multigene Phylogeny of Native American Hawkweeds (Hieracium Subgen. Chionoracium, Cichorieae, Asteraceae): Origin, Speciation Patterns, and Migration Routes
Source: Plants (Basel). 2022 Sep 30;11(19):2584. doi: 10.3390/plants11192584 (PMC9571344; doi:10.3390/plants11192584)
Supplement: Supplementary file 1 [file plants-11-02584-s001.zip › Table S5_marker-characteristics_Plants.pdf]

Table S5: Overview of markers, datasets, and phylogenetic analyses

Marker	No. of characters (coded indels)[1]		No. of parsimony informative characters (%)		BA Model AIC (million generations)	ML Model AIC
ptDNA (combined)	2281	(81)	106	(4.6)	TVM+I+G (1.5)	TVM+F+I+G4
ITS	715	(3)	99	(13.8)	GTR+I+G (2)	GTR+F+I+G4
ETS	575	(7)	92	(16.0)	TVM+G (1)	TVM+F+G4
ITS+ETS (combined)	1290	(10)	191	(14.8)	GTR+I+G (1)	GTR+F+I+G4
5S-NTS	275	(23)	77	(28.0)	TVMef+G (2)	TPM2u+F+G4
gsh1	1182	(142)	234	(19.8)	GTR+I+G (1)	GTR+F+I+G4
gsh1 (paralogs/pseudogenes)	887	(55)	93	(10.5)	TVM+I (2.5)	GTR+F+I+G4
sqs	1408	(102)	291	(20.7)	TVM+I+G (2)	TVM+F+I+G4
combined dataset	6085	(223)	432	(7.1)	GTR+I+G (1)	TIM+F+I+G4

[1] The number of coded indels is included in the number of characters. BA – Bayesian analysis; ML – Maximum Likelihood; AIC – Akaike Information Criterion
